# Prevalence and factors associated with caesarean section in four Hard-to-Reach areas of Bangladesh: Findings from a cross-sectional survey

**DOI:** 10.1371/journal.pone.0234249

**Published:** 2020-06-09

**Authors:** Farhana Karim, Nazia Binte Ali, Abdullah Nurus Salam Khan, Aniqa Hassan, Mohammad Mehedi Hasan, Dewan Md. Emdadul Hoque, Sk. Masum Billah, Shams El Arifeen, Mohiuddin Ahsanul Kabir Chowdhury

**Affiliations:** 1 Maternal and Child Health Division, International Centre for Diarrhoeal Disease Research, Bangladesh (icddr,b), Dhaka, Bangladesh; 2 Arnold School of Public Health, University of South Carolina, South Carolina, United States of America; 3 United Nations Population Fund, Dhaka, Bangladesh; Anglia Ruskin University, UNITED KINGDOM

## Abstract

**Background:**

Caesarean section (C-section) is a major obstetric life-saving intervention for the prevention of pregnancy and childbirth related complications. Globally C-section is increasing, as well as in Bangladesh. This study identifies the prevalence of C-section and socio-economic and health care seeking related determinants of C-section among women living in hard-to-reach (HtR) areas in Bangladesh.

**Methods:**

A cross-sectional survey was conducted using a structured questionnaire between August and December 2017 at four distinct types of HtR areas of Bangladesh, namely coastal, hilly, haor (wetland), and char areas (shallow land-mass rising out of a river). Total 2,768 women of 15–49 years of age and who had delivery within one year prior to data collection were interviewed. For the analysis of determinants of C- section, the explanatory variables were maternal age, educational status of women and their husbands, women’s religion, employment status and access to mobile phone, wealth index of the household, distance to the nearest health facility from the household, the number of ANC visits and presence of complications during pregnancy and the last childbirth. Logistic regression model was run among 850 women, who had facility delivery. Variables found significantly associated with the outcome (C-section) in bivariate analysis were included in the multivariable logistic model. A p-value <0.05 was considered as statistically significant in the analyses.

**Results:**

Of the 2,768 women included in the study, 13% had C-sections. The mean (±SD) age of respondents was 25.4 (± 0.1) years. The adjusted prevalence of C-section was 13.1 times higher among women who had their delivery in private facilities than women who delivered in public facilities (Adjusted Odds Ratio, AOR: 13.1; 95% CI 8.6–19.9; p-value: <0.001). Women from *haor* area and coastal area had 4.7 times (AOR: 4.7; 95% CI 2.4–9.4; p value: <0.001) and 6.8 times (AOR: 6.8; 95% CI 3.6–12.8; p value: <0.001) more chance of having C-section, respectively, than women living in *char* area. Among women who reported complications during the last childbirth, the AOR of C-section was 3.6 times higher than those who did not report any complication (AOR: 3.6; 95% CI 2.4–5.4; p value: <0.001).

**Conclusions:**

The study identifies that the prevalence of C-sections in four HtR areas of Bangladesh in substantially below the national average, although, the prevalence was higher in coastal areas than three other HtR regions. Both public and private health services for C-section should be made available and accessible in remote HtR areas for women with pregnancy complications. Establishment of an accreditation system for regulating private hospitals are needed to ensure rational use of the procedure.

## Background

Caesarean section (C-section), one of the most commonly performed surgical procedures, can be life-saving when complications arise during delivery [1, 2]. C-section has played pivotal role in reducing maternal mortality over the last few decades [2]. Conversely, C-sections without proper indication and justification have several adverse consequences leading to increased maternal and neonatal mortality and morbidity [3]. The global rate of C-section delivery is rising steadily and has reached a rate of 21.1 of all births in 2015 with an average annual increasing rate of 3.7% during 2000–2015 [4]. In south Asia, C-section has doubled during 2000–2015, with average annual increasing rates of exceeding 5% [4]. In this region, C-section rate reached at 18.1% during 2015 exceeding the World Health Organization (WHO) recommended upper limit of C-section rate at 15% of all deliveries [5–7]. The rising rate of C-section indicates that this life saving intervention is being practiced higher than the expected level on the basis of obstetric indications in many countries [4]. C-section can also be costly and places poor families under extreme financial pressure in low and middle income countries (LMIC) [8]. In Bangladesh, the C-section rate has rapidly increased in the last two decades from 3% in 2001 to 33% at population level [9, 10].

Identification of the factors influencing the C-section is critical to minimize the unnecessary practice of such life saving intervention and increase its access to those who needs it the most. Studies showed that factors related to childbearing women, families, communities and the broader society and factors related to health system stimulate the increased demand and supply of C-section related health services [11, 12]. Health care-seeking behaviours such as seeking antenatal care (ANC) [13, 14], occurrence of health complication during pregnancy and labour [15, 16], and types of facility where childbirth takes place, are strongly associated with women having C-section in Bangladesh [14, 17]. In the absence of clinical justification for C-section, there is evidence for women’s personal preference playing crucial role in decision making for C-section [18, 19]. Such individual preference for C-section is found to have link with socio-demographic characteristics of pregnant women such as their age [20–22], education [14, 23], occupation [24], household income and asset [14, 25]. Recent lancet series showed that there were large differences in C-Section use between women in the poorest and the richest wealth quintile in 82 LMICs [4]. Cultural and environmental differences in different geographic areas of a country play a major role in shaping these factors and may also influence the C-section practice in those areas. Bangladesh has diverse geographical features which include remote regions with difficult terrains. Communication is a major challenge in these regions [26]. Each of these areas has distinct characteristics and unique forms of livelihood. Accessibility to different health care services including emergency obstetric care as well as coverage of different key interventions to improve health status of the population is a major challenge in these areas, especially during monsoon due to poor road network and transportation [27].

Several studies explored the determinants of C-section in Bangladesh and other LMICs [14, 16, 28], however, there are very few studies that have explored the utilization and determinants of C-section in geographically isolated or HtR areas. This paper identifies the prevalence of C-section and socio-economic, obstetrics and health care seeking related determinants of C-section among women living in four distinct types of HtR areas in Bangladesh–coastal, hilly, *haor* (wetland), and *char* areas (shallow land-mass rising out of a river. This information can help key stakeholders shape policy on maternal health care services in HtR regions.

## Methodology

### Study design

This paper is a part of an overall bottleneck analysis of Maternal and Neonatal Health (MNH) services in HtR areas of Bangladesh. The bottleneck analysis had a broader objective to explore the availability, accessibility, utilization, and coverage of the MNH care services in HtR areas of Bangladesh. To address the broader study objective, a cross sectional household survey was conducted among recently delivered women (RDW) of 15–49 years of age who had a birth outcome within 12 months prior to the survey. This study uses the data collected from the RDWs during the household survey using a structured questionnaire (S1 Appendix) to calculate and examine the prevalence and determinants of C-sections in the study area.

### Study sites

**Fig 1:**
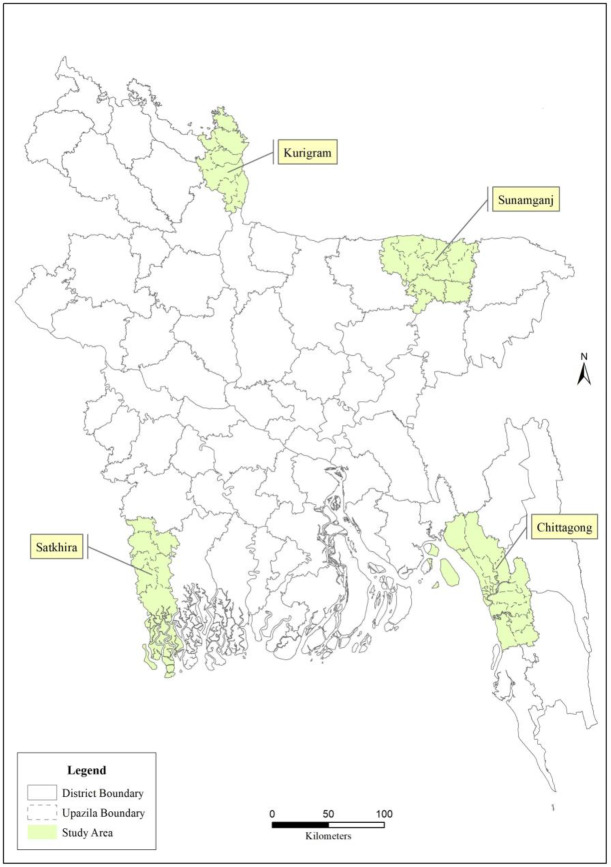
Four study districts (Four HtR areas) of Bangladesh.

The Government of Bangladesh recognises 23 sub-districts in the country as HtR areas. The areas comprise one-fifth of Bangladesh’s total area and are homes to an estimated 29 million people (26). The study sites included four separate HtR areas i) coastal areas (usually lie about 1.5–11.8 meters above the mean sea level) in the Southern region, ii) hill tracts in South-Eastern regions, iii) *haors (*a saucer- shaped shallow depression) in the North-Eastern region, and iv) *char* areas (vegetated islands within river banks) in Northern region of Bangladesh (Fig 1). In this study, total of four HtR districts had been selected and those were Satkhira (coastal), Chittagong (Hilly), Sunamganj (Haor) and Kurigram (Char) (Fig 1). In all four study areas, accessibility is very difficult due to their unique geographical characteristics; most of the regions are rural. In *Haor*, floods take place in every monsoon, while *Chars* are prone to frequent flooding and erosion [29]. The coastal areas are subject to flooding in every monsoon season and water logging in the basin areas during dry season [30] and in hilly areas rugged topography leading to frequent climate change makes communication very hard [31].

### Sample size and sampling

The initial sample size was calculated to address the study objective of bottleneck analysis of MNH services. Using the prevalence of skilled birth attendance at 37% (highest among the four districts), 2616 number of RDWs were required with 5% level of significance, 5% margin of error and 95% confidence interval. Four HtR areas were selected by stratified random sampling from a list districts in each of the four HtR areas. Then, three unions were randomly selected from each of the selected districts. We considered each union as a cluster that had approximately 5000–7000 households. We interviewed a total 2768 RDWs who had delivery within one year of data collection period (*Char*: 691; Hilly: 695; *Haor*: 693 and Coastal: 689). A household listing was conducted in the selected unions to identify the respondents. After completing the desired number of interviews from a union, the household listing was stopped for that union and moved to the next union.

### Definition of the variables

The main outcome variable for this analysis was C-section. The explanatory variables used in this study were selected based on a review of the literature. The socio-demographic variables selected were maternal age, women’s and husband’s educations, religion, employment status, wealth index of the household, four different HtR areas and distance to the nearest health facility from the household. Obstetric health and health care seeking behaviour related factors were the number of ANC visits and health complications during pregnancy or childbirth. Maternal age was categorized into 15–19 years, 20–29 years, and 30–39 years. Education was categorized into ‘no formal education’, 'primary completed or below (1–5)’ and ‘higher than primary’. A household wealth index was calculated using an adapted questionnaire used in the Bangladesh Demographic Health Survey (BDHS) 2014 [32]. The wealth index was created in two steps. First, a composite index of a household's cumulative living standards was calculated by principal component analysis (PCA) from household's ownership of selected assets during the survey. The PCA score was then categorized into five categories- poorest, poorer, middle, richer and richest. Information about distance from home to the nearest health facility providing obstetric health services was collected from the respondents according to their perception of the distance in kilometre (km). The distance reported by the respondents was categorized into three groups; less than 1 km, 1–5 km, and more than 5 km. The number of ANC visits was classified into four mutually exclusive categories. Places of delivery included the home, public health facilities, and private health facilities. The respondents were asked about any complications during the last pregnancy such as the presence of severe headache, blurred vision, oedema of face, legs or hands and presence of convulsion or fits during pregnancy, and malpresentation and prolong labour (more than 12 hours) during labour and childbirth. No clinical documents were checked to verify the respondents’ reports of such health complications during pregnancy and childbirth.

### Data collection and data quality

Survey data collection continued between August and December 2017. A total of 20 data collectors, with previous household survey administration experiences, were trained and deployed to conduct the surveys. A structured validated pretested questionnaire in local language was used for data collections. Extensive training was provided on the different sections of the questionnaire. To reduce any kind of variations among data collectors in collecting data, standardization of the definition of the variables was done and explained to the data collectors. Data collectors visited the respondents’ households and conducted interviews after obtaining written informed consent. Interviewers explained the objectives of the study, expectations from respondents, and the risks and benefits of the study. They were also informed that participation in this study was completely voluntary and they could stop at any time without any obligation during the interview. Supervisors closely monitored the data collection team to ensure completeness and consistency in data collection. They also engaged local people as data collectors to identify the local dialect of words in the questionnaire and give feedback before starting the interview in a new district. For quality assurance, standard protocols were established for all data collection procedures. Moreover, investigators frequently made field visits and review meetings were conducted among data collectors in the presence of study investigators to provide necessary feedback.

### Statistical analysis

Statistical analysis was performed using Stata version 13.0. Simple descriptive statistic such as proportion was used to examine the distribution of samples across different explanatory variables. Findings from descriptive analysis were reported using weighted proportions. For model building, we considered only those respondents whose delivery was conducted in health facilities, excluding the home deliveries. Simple logistic regressions were performed to estimate associations of each explanatory variable with the outcome variable, which was C-section. Variables that showed significant association in simple logistic analysis, were later included in multivariable logistic regression model. Socio-demographic variables (maternal age group, maternal education and wealth index) were adjusted in the multivariable logistic regression model, followed by inclusion of variables found significant in bivariate analyses. Adjusted odds ratios (AOR) with 95% confidence intervals (CI) were reported. A p-value <0.05 was considered statistically significant.

### Ethical consideration

The Ethical Review Committee of icddr,b has reviewed and approved the study (protocol number 17033). Participants provided written informed consent to participate in the study either by signing or by giving a thumbprint (if illiterate). A signature or left thumb impression was also obtained from a witness during the consent process. In the case of participants under the age of 18 years, informed consent was obtained from legal guardians and assent was taken from the participants. The privacy, anonymity, and confidentiality of information were maintained in due process. Auditory privacy was maintained during the interview. All the hardcopy data forms were de-identified by separating the identification page. Unique alphanumeric code was assigned to each data form and entered into the database. The identification page and consent form were kept in lock and key.

## Results

### Socio-demographic and obstetric characteristics of women

A total of 2768 women were interviewed within 12 months of the last birth outcome. The prevalence of C-section at the population level was 13%, although in the coastal area this prevalence was 34% while in other three HtR areas, this prevalence was below 10% (char: 8%; hilly: 9% and haor: 8%). The mean (±SD) age of the respondents was 25.4 (± 0.1) years and about 61% of the women were 20–29 years old. Nearly half of them (49%) completed “higher than primary” level education and 20% of them had C-section. More than one third (35%) women’s husbands had “higher than primary” level of education, and C-section rate was higher (23%) among them than those women’s husbands having only “primary complete or below” level education or no formal education. Prevalence of C-section was increased as family’s economic condition improved; 30% women belonging to the highest wealth quintile had C-section in their most recent delivery. Similarly, the prevalence of C-section was increasing with the increasing number of ANC visits, 15% women had C-section who had at least four ANC visits. Among the women who had their deliveries at private facilities, 74% of them chose C-section, while 24% women choose C-section as their mode of delivery who delivered in public facilities. About 78% women did not report any pregnancy complication and 12% of them had C-section. About 9% women who had no complication during childbirth had C-section (Table 1).

**Table 1:** Socio-demographic and obstetric background, N = 2768.

	Total, N (%*)	NVD, n(%*)	CS, n(%*)
	**2768**	**2404 (86.8)**	**364 (13.2)**
**Maternal age**			
**Mean age ± SD**	25.42 ± 0.11	25.54 ± 0.12	24.70 ± 0.27
15–19	409(14.8)	347(84.7)	62(15.3)
20–29	1680(60.7)	1457(86.7)	224(13.3)
30–39	678(24.5)	600(88.5)	78(11.5)
**Maternal education**			
No formal education	439(15.8)	421(96.0)	18(4.0)
Primary complete or below (1–5)	978(35.3)	905(92.5)	73(7.5)
Higher than primary	1351(48.8)	1079(79.8)	273(20.2)
**Husband’s education**			
No formal education	837(30.2)	796(95.1)	41(4.9)
Primary complete or below (1–5)	940(33.9)	841(89.5)	99(10.5)
Higher than primary	966(34.9)	742(76.8)	224(23.2)
Missing	26(0.9)		
**Maternal occupation**			
Employed	174(6.3)	143(82.5)	30(17.5)
Unemployed	2594(93.7)	2261(87.1)	334(12.9)
**Maternal religion**			
Muslim	2481(89.6)	2178(87.8)	303(12.2)
Non-muslim	287(10.4)	226(78.8)	61(21.2)
**Wealth index**			
Poorest	598(21.6)	571(95.6)	26(4.4)
Poorer	580(20.9)	538(92.8)	42(7.2)
Middle	555(20.0)	507(91.3)	48(8.7)
Higher	525(19.0)	429(81.8)	96(18.2)
Highest	511(18.5)	359(70.2)	152(29.8)
**Woman who owned a mobile phone**			
Yes	1419(51.3)	1177(82.9)	242(17.1)
No	1234(44.6)	1116(90.4)	118(9.6)
Missing	115(4.2)		
**Distance to nearest health facility from home**			
Less than 1 km	1505(54.4)	1266(84.1)	239(15.9)
1–5 km	1128(40.7)	1011(89.7)	117(10.3)
More than 5 km	136(4.9)	127(93.7)	9(6.3)
**Number of Antenatal care visit**			
One ANC visit	438(15.8)	411(93.8)	27(6.2)
Two ANC visits	422(15.2)	367(87.1)	55(12.9)
Three ANC visits	456(16.5)	390(8.7)	65(14.3)
At least four ANC visits	1453(52.5)	1236(85.0)	217(15.0)
**HtR Areas**			
Char	739(26.7)	680(92.0)	59(8.0)
Hilly	749(27.1)	680(90.8)	69(9.2)
Haor	771(27.9)	710(92.1)	61(7.9)
Coastal	509(18.4)	334(65.6)	175(34.4)
**Place of delivery**			
Home delivery	1987(71.8)	1987(100.0)	0 (0.0)
Public Hospital	428(15.5)	327(76.5)	101(23.5)
Private Hospital	354(12.8)	90(25.5)	263(74.4)
**Any complication during last pregnancy**			
Yes	603(21.8)	502(83.3)	101(16.7)
No	2165(78.2)	1902(87.9)	263(12.1)
**Any complication during last childbirth**			
Yes	525(19.0)	353(67.3)	172(32.7)
No	2243(81.0)	2051(91.4)	192(8.6)

* Weighted by survey weight and population size of hard to reach areas

Table 2 summarises the findings of unadjusted OR of C-section at health facilities after controlling for possible covariates in multivariable logistic regression. A total of 850 women had delivered in a health facility. In bivariate analysis, the unadjusted OR of C-section was 2.2 times higher among women whose husbands had higher than primary level education than those who had no formal education (UOR: 2.2; 95% CI 1.4–3.3; p value: <0.001). The unadjusted OR of C-section was 2.4 times higher among those who were employed than those who were not (UOR: 2.4; 95% CI 1.3–4.6; p value: 0.006). Among women who own a mobile phone, the unadjusted OR of having C-section was 1.3 times higher than those who did not use mobile phone (UOR: 1.3; 95% CI 1.0–1.9; p value: 0.025). Unadjusted odds of having C-section was 2.0 times higher among those who had attended at least four or more ANC visits than those who had only one ANC visit (UOR: 2.0; 95% CI 1.2–3.3; p value: 0.004). In bivariate analysis, unadjusted OR of C-section were 9.5 times higher in private health facility than women who had delivery at public health facility (UOR: 9.5; 95% CI 6.7–13.2; p value: <0.001). Women from the hilly region had lower odds of having C-section than women from *char* region (UOR: 0.5; 95% CI 0.3–0.8; p value: 0.006). Women with complications during childbirth were 2.6 times more likely to deliver by C-section than those who did not have such complications (UOR: 2.6; 95% CI 1.9–3.5; p value: <0.001).

**Table 2:** Bivariate analysis of C-section with explanatory variables, N = 850.

Characteristics	UOR^1^	CI (95%)	p value
**Maternal age**			
15–19	1	-	-
20–29	0.92	0.63–1.36	0.691
30–39	1.07	0.67–1.70	0.776
**Maternal education**			
No formal education	1	-	-
Primary complete or below (1–5)	0.95	0.49–1.85	0.888
Secondary and above	1.61	0.87–2.99	0.130
**Husband’s education**			
No formal education	1	-	-
Primary complete or below (1–5)	1.44	0.91–2.28	0.120
Secondary and above	2.16	1.41–3.29	<0.001
**Maternal occupation**			
Unemployed	1	-	
Employed	2.44	1.29–4.60	0.006
**Maternal religion**			
Muslim	1	-	-
Non Muslim	1.01	0.69–1.47	0.952
**Wealth index**			
Lowest	1	-	-
Second	0.93	0.49–1.76	0.819
Middle	0.78	0.39–1.32	0.287
Fourth	1.33	0.75–2.37	0.330
Highest	1.65	0.95–2.87	0.078
**Woman is the main user of mobile phone**			
No	1	-	-
Yes	1.34	1.04–1.89	0.025
**Distance to nearest health facility from home**			
Less than 1 km	1	-	-
1–5 km	0.85	0.63–1.15	0.300
More than 5 km	0.53	0.23–1.23	0.139
**Number of Antenatal care visit**			
One ANC visit	1	-	-
Two ANC visits	1.46	0.81–2.63	0.197
Three ANC visits	1.18	0.67–2.06	0.632
At least four ANC visits	2.00	1.20–3.31	0.004
**HtR Areas**			
Char	1		
Hilly	0.52	0.33–0.83	0.006
Haor	0.76	0.47–1.23	0257
Coastal	1.49	0.97–2.27	0.066
**Place of delivery**			
Public Hospital	1	-	-
Private Hospital	9.52	6.86–13.21	<0.001
**Any complication during last pregnancy**			
No	1	-	-
Yes	1.35	0.97–1.86	0.074
**Any complication during last childbirth**			
No	1	-	-
Yes	2.61	1.93–3.52	<0.001

^1^ The estimates are cluster-adjusted

### Determinants of C-section

After inclusion of all significant variables in multivariable regression model, there was significant association of the HtR areas, place of C-section and presence of reported complications during the last childbirth, with C-section (Table 3). Socio-demographic variables (maternal age group, maternal education and wealth index) were adjusted in the multivariable model. In the final multivariable regression model, odds of C-section was 13.1 times higher among women who had their delivery in private health facilities than women who had their delivery in public health facilities (AOR: 13.1; 95% CI 8.6–19.9; p value: <0.001). Among women who reported complications during the last childbirth, the odds of C-section was 3.6 times higher than those who did not report any complication (AOR: 3.6; 95% CI 2.4–5.4; p value: <0.001). Odds of C-section delivery was 6.8 times higher in coastal region and 4.7 times higher in haor region than char region (AOR: 6.8; 95% CI 3.6–12.8; p value: <0.001 and AOR: 4.7; 95% CI 2.4–9.3; p value: <0.001, respectively). Husband’s education, women’s occupation, women’s ownership on mobile phone, number of ANC visit and having reported complications during last pregnancy were not significantly associated with C-section in the final regression model.

**Table 3:** Determinants of C-section, N = 850.

Characteristics	AOR^1^	CI (95%)	p value
**Maternal age**			
15–19	1	-	-
20–29	1.01	0.61–1.67	0.977
30–39	1.18	0.63–2.21	0.607
**Maternal education**			
No formal education	1	-	-
Primary complete or below (1–5)	0.54	0.22–1.30	0.171
Higher than primary	0.70	0.29–1.69	0.432
**Husband’s education**			
No formal education	1	-	-
Primary complete or below (1–5)	1.04	0.57–1.90	0.888
Higher than primary	1.34	0.74–2.43	0.329
**Maternal occupation**			
Unemployed	1	-	-
Employed	1.70	0.78–3.72	0.179
**Wealth index**			
Lowest	1	-	-
Second	1.19	0.52–2.72	0.676
Middle	0.72	0.32–1.60	0.417
Fourth	1.34	0.61–2.95	0.470
Highest	1.07	0.48–2.37	0.872
**Woman who owned a mobile phone**			
No	1	-	-
Yes	1.10	0.74–1.64	0.637
**Number of Antenatal care visit**			
One ANC visit	1	-	-
Two ANC visits	1.04	0.49–2.20	0.917
Three ANC visits	0.77	0.37–1.60	0.489
At least four ANC visits	1.11	0.57–2.15	0.760
**HtR Areas**			
Char			
Hilly	1.71	0.92–3.20	0.091
Haor	4.71	2.37–9.35	<0.001
Coastal	6.76	3.58–12.82	<0.001
**Place of delivery**			
Public Hospital	1	-	-
Private Hospital	13.08	8.59–19.91	<0.001
**Any complication during last childbirth**			
No	1	-	-
Yes	3.64	2.43–5.44	<0.001

^1^ The estimates are cluster-adjusted

## Discussion

In this study, we examined the prevalence and determinants of C-section in four HtR regions of Bangladesh. A prevalence of 13% was calculated, which was lower than the upper limit of the WHO critical threshold of C-section (15%) for any country [5, 6]. Prevalence of C-section was higher among women who delivered at private facilities than those who delivered at public facilities and among women who had complications during the last childbirth than those who did not. Coastal region had the highest (34%) prevalence of C-section than other three HtR areas. Socio-demographic factors such as women’s religion, education, and occupation, husband’s education, household’s wealth quintile, whether woman was the owner of mobile or not and obstetric factors such as the number of ANC visits and presence of any complications during pregnancy were not significantly associated with C-section.

The prevalence of C-section at 13.2% in HtR found in this study was (much lower than the overall C-section rate reported in Bangladesh Demographic Health Survey 2017 (33%) and Bangladesh Maternal Mortality Survey 2016 (31%) [10, 33]. This could be possibly explained by the geographical characteristics of the HtR areas where access to health services is a major challenge which limits the utilization of basic maternal health services. The population in this study were inhabitants of rural remote area and health care services were difficult to access due to poor road network and transportation [34]. Additionally, the shortage of skilled health workforce at HtR areas could be another possible reason of lower rate of C-section in these areas [35]. However, among four distinct types of HtR areas, prevalence of C-section was higher in coastal region (Satkhira), while in haor, hilly and char region this rate is much lower than the national percentage. Further study is needed to explore the reasons of having high C-section in these areas.

Our study found that the odds of having C-section were higher among women who delivered in private facilities than those who delivered in public facilities, consistent with the historical trend of Bangladesh. In 2001–2003, nearly half of the deliveries in private facilities in Bangladesh were done by C-section [9, 36], which is increasing in recent years. In 2014, six out of ten women delivered in a health facility underwent C-section [32]. Contribution of for-profit private organization was increasing in maternal health service provision in last few years in Bangladesh [37, 38]. Lack of regulatory mechanism, availability of incentives as a result of demand side financing (maternal health voucher scheme) and profit sharing inbuilt with the procedures might result in higher prevalence of C-section in recent years with more than 80% deliveries being done by C-sections in private facilities [14, 39]. Over the past two decades, with economic improvement, profit driven private sector is proliferating rapidly in Bangladesh [40]. More deliveries are being conducted in private facilities resulting in inequities in maternal health service utilization [32, 37]. In India, similar situations were found where institutional delivery was increased, driven by sharp increase of childbirth in the private health facilities [41]. One study conducted in Bangladesh showed that financial motives, the urgency to fulfil the required target set by private hospital authorities played an influencing role in performing increased number of C-section by obstetricians [11]. In this regard, there is a need for stringent supervision and audit system for monitoring indications of C-section to control the expanding rate of C-sections in the private facilities [42].

Complications during childbirth were found to have positive association with C- section in this study. Chances of maternal deaths arise from the risk attributable to pregnancy and childbirth related complications along with lower utilization of poor quality health care services [43]. Although the majority of the Bangladeshi women prefer to have home deliveries, recent evidence suggests that women and their families had contingency plan to visit secondary or tertiary health facilities if any complication arises during labour [44, 45]. Hence, establishing more health facilities with better preparedness to treat and manage complicated obstetric cases will improve service utilization in the HtR areas. Contacts during antenatal care may serve as a platform to inform women on danger signs during pregnancy and childbirth and availability of nearby health services. Studies suggested that women having at least one ANC visit are more likely to deliver in a health facility [46, 47]. The Government of Bangladesh has already taken several initiatives to ensure at least four ANC visits for all pregnant women along with midwifery led ANC, delivery and postnatal care service provision, and the coverage of four ANC visits has increased over the past few years [32, 48, 49]. Now it is high time to prioritize quality ANC, particularly focusing on proper counselling during ANC. Our study found that around 10% women without any complication during the last child-birth, had C-section. Policy makers should pay special attention to this issue so that the injudiciary practice of C-section could be prevented in the facilities without any absolute and relative indications. Moreover, policymakers should also emphasize strengthening emergency referral transportation in HtR areas from home to facility is important to ensure proper management like facility delivery, as well as C-section for those who need it but are not getting it.

The study found neither maternal nor husband’s education was significantly associated with C-section, although evidence shows that better-educated women are more like to deliver in health facilities rather than at home [50–53]. Especially in decision making for the place of delivery, better educated women are more empowered to take their own decision [54]. We also did not find any significant association of socio-economic status with C-section in this study and this finding was contradictory to another study conducted in Bangladesh where chance of C-section increased with improved socio-economic status [14]. The poor communication and lack of accessibility to various health services in the HtR areas might have undermined shadowed the influence of education and wealth on C-section rate in this study.

The main strength of the study was its large sample size from underserved HtR areas of Bangladesh. This study might help policymakers to identify the influencing factors of C-section in HtR areas which is often missing in nationally representative surveys and would help them in targeted programming. This study, however, had a few limitations. It was not a nationally representative study and respondents of this study were only inhabitants of HtR areas. This limits the generalisability of our findings to geographical settings outside the study areas, however, for HtR areas the study provides a robust sample size. Another limitation was poor recall or pregnancy and child birth related events by the respondents because we interviewed those women who had their delivery within one year prior to the data collection period. This might resulted in slight inaccuracy of data reporting as the reported information was not cross-validated with clinical documents. This method of collecting pregnancy and birth related information from the respondents is widely used large nationally representative surveys in LMICs where clinical documentations are poor.

## Conclusions

C-section is an effective intervention to save the lives of mothers and newborns at the time of complications during childbirth. The study identifies that the prevalence of C-sections in four HtR areas of Bangladesh in substantially below the national average, although, the prevalence was higher in coastal areas than three other HtR regions. Both public and private health services for C-section should be made available and accessible in remote HtR areas for women with pregnancy complications. To increase the effective use of C-section evidence-based interventions like use of partograph for monitoring progress of labour should be strengthened [55]Hospitals are needed to be equipped with updated protocol for labour and delivery management and monitoring mechanism needs strengthening for ensuring adherence to the guideline. Routine clinical audits using validated tools are needed to be incorporated into the monitoring system for identifying unnecessary C-sections [56]. Finally, it is important to establish an accreditation system for regulating private hospitals so that C-section is not used for profit [57].

## Supporting information

S1 Appendix Structured questionnaire for recently delivered women. (DOC)
