# Supplementary material for: Prevalence and factors associated with caesarean section in four Hard-to-Reach areas of Bangladesh: Findings from a cross-sectional survey
Source: PLoS One. 2020 Jun 9;15(6):e0234249. doi: 10.1371/journal.pone.0234249 (PMC7282647; doi:10.1371/journal.pone.0234249)
Supplement: S1 Appendix — (DOC) [file pone.0234249.s001.doc]

**S1 Appendix: Structured questionnaire for recently delivered women**

| **m¤cÖwZ cÖmeKvix gwnjv‡`i Rb¨ cÖkœcÎ** Questionnaire for Recently Delivered Women (RDW) | | | | | | | | | | | | | | | |  |
| --- | --- | --- | --- | --- | --- | --- | --- | --- | --- | --- | --- | --- | --- | --- | --- | --- |
|  | **mbv³KiY** (Identification) | | | | | | | | | | | | | | | |
|  |  | | | **bvg** Name | | | | | | **‡KvW** Code | | | | মহিলার মোবাইল নাম্বারঃ Mobile number of RDW  ______________________________________  মহিলার স্বামীর/ আত্মীয়ের/ প্রতিবেশীর মোবাইল নাম্বারঃ Mobile number of RDW’s husband/ relative/ neighbor  _______________________________ | | |
|  | **‡Rjv**  District | | |  | | | | | |  | | | |  |  |  |
|  | **Dc‡Rjv**  Sub-district | | |  | | | | | |  | | | |  |  |  |
|  | **BDwbqb**  Union | | |  | | | | | |  | | | |  |  |  |
|  | **MÖv‡gi bvg I †KvW**  Name of village & code | | |  | | | | | |  | | | |  |  |  |
|  | **evwoi bvg I bs**  Name of the house & number | | |  | | | | | |  | | | |  |  |  |
|  | **Lvbv cÖav‡bi bvg I Lvbv bs**  Name of household head & HH # | | |  | | | | | |  | | | |  |  |  |
|  | **m¤cÖwZ cÖmeKvix gwnjvi bvg I b¤^i** Name of RDW & number | | |  | | | | | |  | | | |  |  |  |
|  | **m¤cÖwZ cÖmeKvix gwnjvi ¯^vgxi bvg**  Name of husband of RDW | | |  | | | | | |  | | | |  |  |  |
|  | **mv¶vrKviMÖnbKvixi cwi`k©b Ges eZ©gvb Ae¯’v** (Interviewer’s visit and status) | | | | | | | | | | | | | | | |
|  |  | | **cwi`k©b-1** Visit-1 | | | **cwi`k©b-2** Visit-2 | **cwi`k©b-3** Visit-3 | | | | | **‡kl cwi`k©b** Final Visit | | | | |
|  | ZvwiL Date | | __ __/ __ __/ __ __ | | | __ __/ __ __/ __ __ | __ __/ __ __/ __ __ | | | | | ZvwiL Date __ __/ __ __/ __ __ | | | | |
|  | **mv¶vZKvi ïiæ nevi mgq:**  Interview starting time: | | |______|______| : |______|______|  N›Uv (Hour) wgwbU (Min) | | | |______|______| : |______|______|  N›Uv (Hour) wgwbU (Min) | |______|______| : |______|______|  N›Uv (Hour) wgwbU (Min) | | | | |  | | |  | |
|  | **mv¶vZKvi †kl Kivi mgq:**  Interview end time: | | |______|______| : |______|______|  N›Uv (Hour) wgwbU (Min) | | | |______|______| : |______|______|  N›Uv (Hour) wgwbU (Min) | |______|______| : |______|______|  N›Uv (Hour) wgwbU (Min) | | | | |  | | |  | |
|  | mv¶vrKviMÖnbKvixi bvg Name of Data Collector | |  | | |  |  | | | | | mv¶vZKviMÖnbKvixi †KvW (DC code) | | | |_____|_____|_____|_____| | |
|  | Result code* | | |_____|_____| | | | |_____|_____| | |_____|_____| | | | | | **‡iRvë †KvW***  Result code* | | | |_____|_____| | |
|  | cieZ©x cwi`k©b  Next visit | | **ZvwiLt**  Date | | | **ZvwiLt**  Date |  | | | | | **‡gvU cwi`k©b**  Total # of visit | | | |_____| | |
|  |  |  | **mgqt**  Time | | | **mgqt**  Time |  |  |  |  |  |  |  |  |  |  |
|  | RESULT CODES*: | | | | | |  | | |  | | | | | | |
|  | 01. | **B›UviwfD mgvß** Interview Complete | | | | | | | 06. | | **m¤cÖwZ cÖmeKvix gwnjv Abycw¯’Z** RDW is absent | | | | | |
|  | 02. | **evwo cwi`k©‡bi mgq Lvbvi †Kvb m`m¨‡K ev Dchy³ KvD‡K cvIqv hvq bvB**  No household member or competent respondent were present at home at time of household visit | | | | | | | 07. | | **MZ** 1 RyjvB 2016 †_‡K 30 Ryb 2017 **mg‡qi g‡a¨ GB Lvbvi †Kvb gwnjvi Mf© †kl nq bvB** No woman with a pregnancy outcome during 1/7/2016 to 30/6/2017 | | | | | |
|  | 03. | **B›UviwfD evwZj** Interview cancelled | | | | | | |  | |  | | | | | |
|  | 04. | **B›UviwfD w`‡Z ivRx bq** Refused to give interview | | | | | | | 08. | | **Ab¨vb¨** Others _____________________________________________ | | | | | |
|  | 05. | **evm¯’vbwU Luy‡R cvIqv hvq bvB** Could not find the residence | | | | | | |  | | (D‡jøL Kiæb) specify | | | | | |
|  |  | | | | | | | | | | | | | | | |
|  | (Supervision) **ZË¡veavqb** | | | | (Name) **bvg** | | | (Code) **†KvW** | | | | | (Date) **ZvwiL** | | | |
|  | Reviewed by Field Editor | | | |  | | | |____|____|____|____| | | | | | |____|____|-|____|____|-|____|____| | | | |
|  | Checked by Supervisor | | | |  | | | |____|____|____|____| | | | | | |____|____|-|____|____|-|____|____| | | | |

**Section A: Household Section (Lvbvi Z_¨)**

**GLb Avwg Avcbvi Ges Avcbvi Lvbvi m¤ú‡K© wKQz Z_¨ Rvb‡Z PvB|** Now I would like to know some information about you and your household.

| **No.** | **Questions** | **Responses** | | | | | **Code** | | | | | **skip** | |
| --- | --- | --- | --- | --- | --- | --- | --- | --- | --- | --- | --- | --- | --- |
| 11 | **Avcbvi Lvbvq mvaviYZ KZRb †jvK evm K‡i? A_©vr Avcbv‡`i Lvbvi †gvU m`m¨ msL¨v KZRb?**  How many members usually live in your household? Please give the number of household member | **Lvbvi †gvU m`m¨ msL¨v**  Total # of household member | | | | |  | | | | |  | |
| 12 | **Avcbv‡`i Lvbvq †Kvb& eq‡mi KZRb cyiæl Ges gwnjv Av‡Q ejyb**  Now tell me how many male and female members are there in your household:  **‡Kvb eq‡mi cyiæl Ges gwnjv m`m¨ bv _vK‡j e‡·** ‘00’ **wjLyb|**  If none, write ‘00’ in box.  ***(cyiæl Ges gwnjvi msL¨v †hvM K‡i †gvU Gi e‡· wjLyb)***  Calculate the total number of male and female and write in the Total box |  | | | **cyiæl**  Male | | **gwnjv**  Female | | | | | |  |
|  |  | 0-4 **eQi** Years | | |  | |  | | | | | |  |
|  |  | 5-14 **eQi** Years | | |  | |  | | | | | |  |
|  |  | 15-29 **eQi** Years | | |  | |  | | | | | |  |
|  |  | 30-49 **eQi** Years | | |  | |  | | | | | |  |
|  |  | 50 **eQi** **ev †ekx** ≥50 Years | | |  | |  | | | | | |  |
|  |  | **‡gvU** Total | | |  | |  | | | | | |  |
| 13 | **mv¶vrKviMÖnYKvixt cÖkœ 12 †_‡K cyiæl Ges gwnjvi †gvU msL¨v e‡· wj‡L cÖkœ 11 Gi mv‡_ wgwj‡q †`Lyb| AmvgÄm¨ n‡j cÖ‡qvR‡b cÖkœ 11 Ges/ev cÖkœ 12 ms‡kvab Kiæb|**  Write the total of male and female members from Q12 in the box and reconcile with Q11 | **‡gvU cyiæl Ges gwnjv**  Total number of male and female | | | | |  | | | | |  | |
| 14 | **_vjv evmb †avqvi Rb¨ cÖavbZt Avcbviv †Kv_vKvi cvwb e¨envi K‡ib?**  What is the main source of water your household used for cleaning utensils? | **cvB‡ci cvwb Piped water:** | | | | | | | |  | |  | |
|  |  | **evwoi wfZ‡i U¨v‡ci cvwb** Piped inside dwelling | | | | | | | | 11 | |  | |
|  |  | **evwoi evwn‡i U¨v‡ci cvwb** Piped outside dwelling | | | | | | | | 12 | |  | |
|  |  | **K~‡ci cvwb Well water:** | | | | | | | |  | |  | |
|  |  | **bjKzc** Tubewell | | | | | | | | 21 | |  | |
|  |  | **AMfxi bjK~c** Shallow Tubewell | | | | | | | | 22 | |  | |
|  |  | **Mfxi bjK~c** Deep Tubewell | | | | | | | | 23 | |  | |
|  |  | **K~qv** Surface Well/Other Well | | | | | | | | 24 | |  | |
|  |  | **f~-c„‡ôi cvwb Surface water:** | | | | | | | |  | |  | |
|  |  | **cyKzi/e× Rjvkq/n«`** Pond/Tank/Lake | | | | | | | | 31 | |  | |
|  |  | **b`x/Lvj/SY©vi cvwb** River/Stream | | | | | | | | 32 | |  | |
|  |  | **e„wói cvwb** Rain water | | | | | | | | 41 | |  | |
|  |  | **Ab¨vb¨** Other________________________  (wbw`©ó Kiæb) Specify | | | | | | | | 96 | |  | |
|  |  |  |  |  |  |  |  |  |  |  |  |  |  |
| 15 | **Avcbvi Lvbvi m`m¨iv mvavibZ wK ai‡Yi cvqLvbv/j¨vwUªb e¨envi K‡i?**  What kind of toilet facility do members of your household usually use? | **d¬vwks Uq‡jU Flush Toilet** | | | | | | | |  | |  | |
|  |  | **d¬vk K‡i ‡mcwUK U¨vs‡KcvVv‡bv / AvaywbK j¨vwUªb**  Flush to Septic Tank/Modern Toilet | | | | | | | | 11 | |  | |
|  |  | MZ© (wcU) Uq‡jU/j¨vwUªb Pit Toilet/Latrine: | | | | | | | |  | |  | |
|  |  | **Rjve×/¯øvve (m¨vwbUvix) j¨vwUªb** Water sealed/Slab Latrine | | | | | | | | 21 | |  | |
|  |  | **Rjve× bq, M‡Z©i (wcU) j¨vwUªb** Pit Latrine | | | | | | | | 22 | |  | |
|  |  | **‡Lvjv/SzjšÍ j¨vwUªb** Open/Hanging Latrine | | | | | | | | 23 | |  | |
|  |  | **j¨vwUªb bvB/†Svc-Svo/gvV** No Facility/Bush/Field | | | | | | | | 31 | |  | |
|  |  | **Ab¨vb¨** Other____________________________  wbw`©ó Kiæb (Specify) | | | | | | | | 96 | |  | |
|  |  |  |  |  |  |  |  |  |  |  |  |  |  |
| 16 | Avcbvi Lvbvq wK wb‡gœi wRwbl¸‡jv e¨envi Dc‡hvMx Ae¯’vq Av‡Q?  Does your household have following usuable goods?  we`¨yr/‡mŠi we`¨yr Electricity/Solar electricity?  ‡iwWI A radio?  †Uwjwfkb A television  †gvevBj †dvb A mobile phone?  †gvevBj Qvov Ab¨ †Uwj‡dvb A non-mobile phone?  wd«R A refrigerator  Avjgvix/IqvW©‡ive An almirah/wardrobe?  ‡Uwej A table?  ‡Pqvi/†eÂ A chair?  ‰e`¨wZK cvLv An electric fan?  wWwfwW/wfwmwW †cøqvi A DVD/VCD player?  cvwbi cv¤ú A water pump?  wiKmv/f¨vb Rickshaw/Van  evBmvB‡Kj Bicycle  †gvUimvB‡Kj Motorcycle  wm,Gb,wR/†U¤úy/B‡jKwUªK evBK CNG/Tempo/Electric bike  ‡bŠKv Boat  AvB, wc, Gm/‡Rbv‡iUi IPS/Generator  KgwcDUvi/j¨vcUc Computer/Laptop  cÖ‡Z¨KwU wRwbm m¤^‡Ü wR‡Ám Kiæb  *Ask for every item* |  | **ITEM** | | | YES | | NO | | | **No.**  **msL¨v** |  | |
|  |  | A | we`¨yr Electricity | | | 1 | | 2 | | |  |  | |
|  |  | B | ‡mŠi we`¨yr/ Solar electricity | | | 1 | | 2 | | |  |  | |
|  |  | C | ‡iwWI radio | | | 1 | | 2 | | |  |  | |
|  |  | D | †Uwjwfkb television | | | 1 | | 2 | | |  |  | |
|  |  | E | †gvevBj †dvb mobile phone | | | 1 | | 2 | | |  |  | |
|  |  | F | Ab¨ †Uwj‡dvb non-mobile phone | | | 1 | | 2 | | |  |  | |
|  |  | G | wd«R refrigerator | | | 1 | | 2 | | |  |  | |
|  |  | H | Avjgvix/IqvW©‡ive/‡kv‡Km  almirah/wardrobe | | | 1 | | 2 | | |  |  | |
|  |  | I | ‡Uwej table | | | 1 | | 2 | | |  |  | |
|  |  | J | ‡Pqvi/†eÂ chair/bench | | | 1 | | 2 | | |  |  | |
|  |  | K | ‰e`¨wZK cvLv electric fan | | | 1 | | 2 | | |  |  | |
|  |  | L | wWwfwW/wfwmwW †cøqvi DVD/VCD player | | | 1 | | 2 | | |  |  | |
|  |  | M | cvwbi cv¤ú water pump | | | 1 | | 2 | | |  |  | |
|  |  | N | wiKmv/f¨vb Rickshaw/Van | | | 1 | | 2 | | |  |  | |
|  |  | O | evBmvB‡Kj Bicycle | | | 1 | | 2 | | |  |  | |
|  |  | P | †gvUimvB‡Kj Motorcycle | | | 1 | | 2 | | |  |  | |
|  |  | Q | wm,Gb,wR/†U¤úy/B‡jKwUªK evBK CNG/Tempo/Electric bike | | | 1 | | 2 | | |  |  | |
|  |  | R | ‡bŠKv Boat | | | 1 | | 2 | | |  |  | |
|  |  | S | AvB, wc, Gm/‡Rbv‡iUi IPS/Generator | | | 1 | | 2 | | |  |  | |
|  |  | T | KgwcDUvi/ ল্যাপটপ  Computer/Laptop | | | 1 | | 2 | | |  |  | |
| 17 | GB Lvbvi wbR¯^ KZ¸wj wb‡gœ ewY©Z cï-cvwL Av‡Q?  How many of the following animals does this household own?  Rvbv bv _vK‡j ‘97’ wjLyb If unknown, enter ‘97’  ‡KvbwU bv _vK‡j ‘00’ wjLyb If none enter ‘00’  95 wU ev Zvi †ekx _vK‡j ‘95’ wjLyb If 95 or more, enter ‘95’  gwnl Buffaloes?  lvuo/Miæ Bulls/ Milk cows?  QvMj/†fov Goat / sSeep?  gyiwM/nvum Chicken / Duck? |  | **cïcvwL**  **Animal** | | | | | | **msL¨v Number** | | |  | |
|  |  | A | gwnl Buffaloes | | | | | |  | | |  | |
|  |  | B | lvuo / Miæ Bulls / Milk cows | | | | | |  | | |  | |
|  |  | C | QvMj / †fov Goat / shee | | | | | |  | | |  | |
|  |  | D | gyiwM / nvum Chicken / Duck | | | | | |  | | |  | |
| 18 | **emZ N‡ii Pv‡ji/Qv‡`i cÖavb wbg©vY-mvgMÖxt**  **(‡`‡L wjwce× Kiæb)**  Main Material of the **Roof:**  (Record observation.) | **KuvPv Qv` Natural Roof:** | | | | | | | |  | |  | |
|  |  | **ছন/ Li/cvZv** (Thatch / leaf)) | | | | | | | | 11 | |  | |
|  |  | **cÖv_wgK ch©v‡qi Qv` Rudimentary Roof:** | | | | | | | |  | |  | |
|  |  | evuk Bamboo | | | | | | | | 21 | |  | |
|  |  | **Kv‡Vi Z³v** Wood planks | | | | | | | | 22 | |  | |
|  |  | **cwic~Y© Qv` Finished roof:** | | | | | | | |  | |  | |
|  |  | **wUb**  Tin | | | | | | | | 31 | |  | |
|  |  | **cwjm Kiv KvV** Polished Wood | | | | | | | | 32 | |  | |
|  |  | **wmivwgK UvBjm** Ceramic tiles | | | | | | | | 33 | |  | |
|  |  | **wm‡g›U, KbwµU** Cement/Concrete | | | | | | | | 34 | |  | |
|  |  | **Ab¨vb¨** Other___________________________  **wbw`©ó Kiæb** (Specify) | | | | | | | | 96 | |  | |
| 19 | **emZ N‡ii †`qv‡ji cÖavb wbg©vY-mvgMÖxt**  **(‡`‡L wjwce× Kiæb)**  Main material of the **Walls**:  (Record observation.) | **KuvPv †`qvj Natural Walls:** | | | | | | | |  | |  | |
|  |  | **†Kvb †`qvj †bB** No walls | | | | | | | | 11 | |  | |
|  |  | **Qwi / cvZv / Mv‡Qi KvÛ** Cane/Palm/Trunks | | | | | | | | 12 | |  | |
|  |  | **cÖv_wgK ch©v‡qi †`qvj Rudimentary Walls:** | | | | | | | |  | |  | |
|  |  | **gvwU** Earth/clay/mud | | | | | | | | 21 | |  | |
|  |  | **evuk** Bamboo | | | | | | | | 22 | |  | |
|  |  | **cv_i** Stone | | | | | | | | 23 | |  | |
|  |  | **Kv‡Vi Z³v** Wood planks | | | | | | | | 24 | |  | |
|  |  | **cwic~Y© †`qvj Finished Walls:** | | | | | | | |  | |  | |
|  |  | **wUb**  Tin | | | | | | | | 31 | |  | |
|  |  | **wm‡g›U** Cement | | | | | | | | 32 | |  | |
|  |  | **BU** Brick | | | | | | | | 33 | |  | |
|  |  | **Ab¨vb¨** Other_________________________ | | | | | | | | 96 | |  | |
|  |  | wbw`©ó Kiæb (Specify) | | | | | | | |  |  |  |  |
| 20 | **emZ N‡ii ‡g‡Si cÖavb wbg©vY-mvgMÖxt**  **(‡`‡L wjwce× Kiæb)**  Main material of the **Floor**:  (Record observation.) | **KuvPv †g‡S Natural floor:** | | | | | | | |  | |  | |
|  |  | **gvwU / evjy** Earth / Sand | | | | | | | | 11 | |  | |
|  |  | **cÖv_wgK ch©v‡qi †g‡S Rudimentary floor:** | | | | | | | |  | |  | |
|  |  | **Kv‡Vi Z³v** Wood planks | | | | | | | | 21 | |  | |
|  |  | **evuk** Bamboo | | | | | | | | 22 | |  | |
|  |  | **cwic~Y© †g‡S Finished floor:** | | | | | | | |  | |  | |
|  |  | **cwjm Kiv KvV** Polished Wood | | | | | | | | 31 | |  | |
|  |  | **wmivwgK UvBjm** Ceramic tiles | | | | | | | | 32 | |  | |
|  |  | **wm‡g›U, KbwµU** Cement/Concrete | | | | | | | | 33 | |  | |
|  |  | **Ab¨vb¨** Other___________________________ | | | | | | | | 96 | |  | |
|  |  | wbw`©ó Kiæb (Specify) | | | | | | | |  |  |  |  |
| 21 | **Avcbv‡`i Lvbvi gvwjKvbvq emZ wfUv Av‡Q wK?**  **hw` bv nq,** PROBE **Kiæbt**  **Avcbv‡`i Lvbvi Ab¨ †Kv_vI emZ wfUv Av‡Q wK?**  Does your household own any homestead?  IF ‘NO’, PROBE:  Does your household own homestead at any other places? | | | **nu¨v** Yes | | | | | | 1 | |  | |
|  |  |  |  | **bv** No | | | | | | 2 | |  |  |
| 22 | **(Lvbvi emZ wfUv Qvov) Avcbv‡`i আর †Kvb Rwg (চাষের/ মাছের ঘের) Av‡Q wK?** Does your household own any land (other than the homestead land)? | | | **nu¨v** Yes | | | | | | 1 | |  | |
|  |  |  |  | **bv** No | | | | | | 2 | |  | |
| 23 | **ivbœv Kivi Rb¨ Avcbvi Lvbvq mvavibZ wK ai‡bi R¡vjvwb e¨envi Kiv nq?**  What type of fuel does your household mainly use for cooking? | **we`¨yr** Electricity | | | | | | | | 01 | |  | |
|  |  | **Gj,wc,wR M¨vm** LPG gas | | | | | | | | 02 | |  | |
|  |  | **cÖvwKwZK M¨vm** Natural gas | | | | | | | | 03 | |  | |
|  |  | **‰Re M¨vm** Bio gas | | | | | | | | 04 | |  | |
|  |  | **‡K‡ivwmb †Zj** Kerosine oil | | | | | | | | 05 | |  | |
|  |  | **LwbR Kqjv** Coal, Lignite | | | | | | | | 06 | |  | |
|  |  | **Ab¨ Kqjv** Charcoal | | | | | | | | 07 | |  | |
|  |  | **KvV** Wood | | | | | | | | 08 | |  | |
|  |  | **Li/KzUv/kyK‡bv Nvm** Straw/Shrubs/Grass | | | | | | | | 09 | |  | |
|  |  | **K…wlRvZ km¨** Agricultural crop | | | | | | | | 10 | |  | |
|  |  | **‡Mvei** Cow dung | | | | | | | | 11 | |  | |
|  |  | **Lvbvq ivbœv Kiv nq bv** No food cooked in household | | | | | | | | 95 | |  | |
|  |  | **Ab¨vb¨** Other (specify)_________________ | | | | | | | | 96 | |  | |

**Section B: RDW and her husband’s background** **m¤cÖwZ cÖmeKvix gwnjv Ges Zvi ¯^vgxi Z_¨**

**GLb Avwg Avcbvi (m¤cÖwZ cÖmeKvix gwnjv) Ges Avcbvi ¯^vgx m¤ú‡K© wKQz cÖkœ wR‡Ám Ki‡Z PvB|**

Now I would like to ask some questions about you (recently delivered woman) and your husband

| **No.** | **Questions** | **Responses & code** | | | **Skip** |
| --- | --- | --- | --- | --- | --- |
| 101 | **Avcwb †Kvb& mv‡ji †Kvb& gv‡m Rb¥MÖnb K‡iwQ‡jb?**  In what month and year were you born? | **gvm** Month | | |  |
|  |  | **Rvwb bv** Don’t know month 97 | | |  |
|  |  | **mvj** Year | | |  |
|  |  | **Rvwb bv** Don’t know year 9997 | | |  |
| 102 | **eZ©gv‡b Avcbvi eqm KZ?** How old are you now?  **(102 Ges 101 wgwj‡q †`Lyb, AmvgÄm¨ n‡j 102 Ges/ev 101 ms‡kvab করুন )**  (Compare and correct 101 and/or 102 if inconsistent) | **eqm (c~Y© eQ‡i)** Age in completed years | | |  |
| 103 | **Avcwb wK KLbI ¯‹z‡j ev gv`ªvmvq †jLvcov K‡i‡Qb?**  Have you ever attended school/madrasa? | **nu¨v** **¯‹zj** Yes School | 1 | |  |
|  |  | **nu¨v** **gv`ªvmv** Yes Madrasa | 2 | |  |
|  |  | **nu¨v** **DfqB** Yes Both | 3 | |  |
|  |  | **bv** No | 4 | | 🡪106 |
| 104 | **Avcwb me©‡kl কোন ch©šÍ¨ covïbv K‡i‡Qb?**  What is the highest level of school you have last attended: primary, secondary, or higher? | **cÖvBgvix/cÖv_wgK** Primary | 1 | |  |
|  |  | **gva¨wgK** Secondary | 2 | |  |
|  |  | **K‡jR/wek¦we`¨vjq** College/university | 3 | |  |
| 105 | **Avcwb m‡e©v”P †Kvb& K¬vk cvk K‡i‡Qb?**  What is the highest class that you have comleted? | **K¬vk** Class  **(†Kvb K¬vk cvk bv Ki‡j** ‘00’ **wjLyb|)**  If completed less than one year, record “00” | | |  |
| 106 | **Avcbvi ag© wK?**  What is your religion? | **Bmjvg** Islam | 1 | |  |
|  |  | **wn›`y** Hinduism | 2 | |  |
|  |  | **‡eŠ×** Buddhism | 3 | |  |
|  |  | **L„óvb** Christianity | 4 | |  |
|  |  | **Ab¨vb¨** **wbw`©ó Kiæb** Other:____________________ | 6 | |  |
| 107 | **Avcwb nqZ Rv‡bb ‡h †Kvb †Kvb gwnjv bM` UvKv ev wRwbmc‡Îi wewbg‡q KvR K‡i, †KD wRwbmcÎ wewµ K‡i, †KD wb‡Ri †QvU e¨emvq ev cvwievwiK Lvgv‡i KvR K‡i| eZ©gv‡b Avcwb G ai‡bi wKQz ev Ab¨ †Kvb KvR Ki‡Qb wK?**  As you know, some women take up jobs for which they are paid in cash or kind. Others sell things, have a small business or work on the family farm or in the family business. Are you doing any of this things or any work? | **nu¨v** Yes | 1 | |  |
|  |  | **bv** No | 2 | | 🡪110 |
| 108 | **cÖavbZt Avcwb wK KvR K‡ib?**  What is your primary occupation, that is, what kind of work do you mainly do?  **wbw`©ó †ckv bx‡P wjLybt**  Write down the occupation below:  _____________________________________  _____________________________________ | **kvixwiK cwikªg wfwËK KvRt Physical work:** | | |  |
|  |  | **wb‡Ri Rwg‡Z Pvlvev` ev eM©vPvlx**  Work on own farm or as a share cropper | 01 | |  |
|  |  | **w`b gRyi/A`¶ kªwgK (M„n¯’vjx,K…wl wfwËK BZ¨vw`)**  Day/unskilled laborer (domestic, agricultural and igrant) | 02 | |  |
|  |  | **`¶ kªwgK (KvVwg¯¿x/ivRwg¯¿x/†R‡j)**  Skilled worker (long term contracted laborer) | 03 | |  |
|  |  | **wiK&mv PvjK/f¨vb PvjK/†bŠKv PvjK**  Rickshaw/Van puller/Boat driver | 04 | |  |
|  |  | **A-kvixwiK cwikªg wfwËK KvRt Non physical work:** |  | |  |
|  |  | **wbR¯^ e¨emv** (†`vKvb/†mjvB/Lvgvi/KzwUi wkí) Own business | 05 | |  |
|  |  | **PvKzixRxwe/†ckvRxwe (Wv³vi, cÖ‡KŠkjx, DwKj, wk¶K)**  Service holder/Professionals | 06 | |  |
|  |  | **Ab¨vb¨** wbw`©ó Kiæb Other: | 96 | |  |
| 109 | **Avcwb wK mviv eQi a‡i KvR K‡ib? bvwK wbw`©ó †gŠmy‡g KvR K‡ib? bvwK gv‡S g‡a¨ KvR K‡ib?**  Do you usually work throughout the year, or do you work seasonally, or only once in a while? | **mviv eQi a‡i** Throughout the year | 1 | |  |
|  |  | **wbw`©ó †gŠmy‡g** Seasonally/Part of the year | 2 | |  |
|  |  | **gv‡S g‡a¨** Once in a while | 3 | |  |
| 110 | **Avcwb eZ©gv‡b weevwnZv, wew”Qbœv, cwiZ¨³v, weaev bv ZvjvKcÖvßv bvwK KLbI we‡q nqwb?**  Are you currently married or separated or deserted or divorced or widowed, or never been married? | **eZ©gv‡b weevwnZv** Currently married | 1 | |  |
|  |  | **wew”Qbœv** Separated | 2 | | 🡪119 |
|  |  | **cwiZ¨³v** Deserted | 3 | | 🡪119 |
|  |  | **ZvjvKcÖvßv** Divorced | 4 | | 🡪119 |
|  |  | **weaev** Widowed | 5 | | 🡪119 |
|  |  | **KLbI we‡q nqwb** Never married | 6 | | **🡪END** |
| 111 | **Avcbvi ¯^vgx †Kvb& mv‡ji †Kvb& gv‡m Rb¥MÖnb K‡iwQ‡jb?**  In what month and year were your husband born? | **gvm** Month | | |  |
|  |  | **Rvwb bv** Don’t know month 97 | | |  |
|  |  | **mvj** Year | | |  |
|  |  | **Rvwb bv** Don’t know year 9997 | | |  |
| 112 | **eZ©gv‡b Avcbvi ¯^vgxi eqm KZ?**  How old is he now?  **(111 Ges 112 wgwj‡q †`Lyb, AmvgÄm¨ n‡j 111 Ges/ev 112 ms‡kvab করুন)**  (Compare and correct 111 and/or 112 if inconsistent) | **eqm (c~Y© eQ‡i)** Age in completed years | | |  |
| 113 | **Avcbvi ¯^vgx KLbI ¯‹z‡j ev gv`ªvmvq †jLvcov K‡i‡Qb wK?**  Has your husband ever attended school /madrasa? | **nu¨v** **¯‹zj** Yes School | | 1 |  |
|  |  | **nu¨v** **gv`ªvmv** Yes Madrasa | | 2 |  |
|  |  | **nu¨v** **DfqB** Yes Both | | 3 |  |
|  |  | **bv** No | | 4 | 🡪116 |
| 114 | **Avcbvi ¯^vgx me©‡kl †Kvb& we`¨vjq/¯Íi ch©šÍ¨ covïbv K‡i‡Qb?**  What is the highest level of school your husband attended: primary, secondary, or higher? | **cÖvBgvix/cÖv_wgK** Primary | | 1 |  |
|  |  | **gva¨wgK** Secondary | | 2 |  |
|  |  | **K‡jR/wek¦we`¨vjq** College/university | | 3 |  |
| 115 | **Avcbvi ¯^vgx m‡e©v”P †Kvb& K¬vk cvk K‡i‡Qb?**  What is the highest class your husband completed? | **K¬vk** Class  **(†Kvb K¬vk cvk bv Ki‡j** ‘00’ **wjLyb|)**  If completed less than one year, record “00” | | |  |
| 116 | **eZ©gv‡b Avcbvi ¯^vgx Avq †ivRMv‡ii Rb¨ †Kvb KvR K‡ib wK?**  Does your husband do anything for living? | **nu¨v** Yes | 1 | |  |
|  |  | **bv** No | 2 | | 🡪119 |
| 117 | **Avcbvi ¯^vgxi cÖavb †ckv wK?**  **GKvwaK †ckvi mv‡_ RwoZ n‡j cÖavb †ckvi bvg wb‡P wj‡L Wvb w`‡Ki †KvW e„ËvwqZ Kiæb|**  What is his primary occupation, that is, what kind of work does he mainly do?  **wbw`©ó†ckv bx‡P wjLybt**  Write down the occupation below:  _____________________________________  _____________________________________ | **kvixwiK cwikªg wfwËK KvRt Physical work:** |  | |  |
|  |  | **wb‡Ri Rwg‡Z Pvlvev` ev eM©vPvlx** Work on own farm or as a share cropper | 01 | |  |
|  |  | **w`b gRyi/A`¶ kªwgK (M„n¯’vjx, K…wl wfwËK BZ¨vw`)** Day/unskilled laborer (domestic, agricultural and migrant) | 02 | |  |
|  |  | **`¶ kªwgK (`xN© †gqv‡` Pyw³e×/ KvVwg¯¿x/ivRwg¯¿x/†R‡j**  Skilled worker (long term contracted laborer) | 03 | |  |
|  |  | **wiK&mv PvjK/f¨vb PvjK/†bŠKv PvjK**  Rickshaw/Van puller/Boat driver | 04 | |  |
|  |  | **A-kvixwiK cwikªg wfwËK KvRt Non physical work:** |  | |  |
|  |  | **wbR¯^ e¨emv** (†`vKvb/†mjvB/Lvgvi/KzwUi wkí) Own business | 05 | |  |
|  |  | **PvKzixRxwe/†ckvRxwe (Wv³vi, cÖ‡KŠkjx, DwKj, wk¶K)**  Service holder/Professionals | 06 | |  |
|  |  | **Ab¨vb¨** wbw`©ó Kiæb Other: ____________________ | 96 | |  |
| 118 | **Avcbvi ¯^vgx wK mviv eQi a‡i KvR K‡ib? bvwK wbw`©ó †gŠmy‡g KvR K‡ib? bvwK gv‡S g‡a¨ KvR K‡ib?** Does your husband usually work throughout the year, or does he work seasonally, or only once in a while? | **mviv eQi a‡i** Throughout the year | 1 | |  |
|  |  | **wbw`©ó †gŠmy‡g** Seasonally/Part of the year | 2 | |  |
|  |  | **gv‡S g‡a¨** Once in a while | 3 | |  |
| 119 | আপনার ঘরে কি মোবাইল ফোন আছে? Do you have mobile phone in your home? | **nu¨v** Yes | 1 | |  |
|  |  | **bv** No | 2 | | 🡪201 |
| 120 | **nu¨v** হলে কয়টি? How many? |  |  | |  |
| 121 | কি ধরনের অপারেটিং সিস্টেম? Type of mobile  (GKvwaK DËi MÖnY‡hvM¨)  (multiple answers acceptable)  (প্রশ্নকর্তা অংশগ্রহণকারীকে বুঝিয়ে বলুন।) | বেসিক Basic | A | |  |
|  |  | এন্ড্রয়েড Android | B | |  |
|  |  | উইন্ডোজ Windows | C | |  |
|  |  | আইফোন iphone | D | |  |
|  |  | অন্যান্য (নির্দিষ্ট করুন) Others _______________________ | Y | |  |
| 122 | মোবাইল ফোনের মূল ব্যবহারকারী কে? Who is the main user?  (GKvwaK DËi MÖnY‡hvM¨)  (multiple answers acceptable)  (প্রশ্নকর্তা অংশগ্রহণকারীকে বুঝিয়ে বলুন।) | আমি নিজে Myself | A | |  |
|  |  | পরিবারের কর্তা/ স্বামী Household head/ husband | B | |  |
|  |  | অন্যান্য (নির্দিষ্ট করুন) Others _______________________ | Y | |  |
| 123 | আপনি কি মোবাইল ফোনের বিভিন্ন ধরণের এপ্লিকেশন চালনা/ ব্যবহার করতে পারেন? Can you operate different mobile application? | **nu¨v** Yes | 1 | |  |
|  |  | **bv** No | 2 | |  |

**Section C: Information about nearest health facility নিকটবর্তী স্বাস্থ্যকেন্দ্র সম্পর্কে তথ্য**

| **No.** | **Questions and filters** | **Responses** | **Code** | **Skip** |
| --- | --- | --- | --- | --- |
| 201 | আপনার বাড়ি থেকে নিকটবর্তী স্বাস্থ্যকেন্দ্র কতদূরে অবস্থিত? How far the nearest helath care center is from your home? | আধা কিমি এর মাঝে < 0.5 km | 1 |  |
|  |  | ০.৫- ১.০ কিমি এর মাঝে within 0.5- 1.0 km | 2 |  |
|  |  | ১.০ -৫.০ কিমির মাঝে within 1.0- 5.0 km | 3 |  |
|  |  | ৫.০ কিমির থেকে বেশি দূরুত্বে >0.5 km | 4 |  |
| 202 | স্বাস্থ্যকেন্দ্রে আপনি কিভাবে যাতায়াত করে থাকেন? How do you ususally gop to nearest health care center? | পায়ে হেটে on foot | 1 | 🡪**205** |
|  |  | যানবাহন by vehicle | 2 |  |
|  |  | উভয়ই both | 3 |  |
| 203 | ¯^v¯’¨‡K‡›`ª hvIqvi Rb¨ Avcbv‡K wK wK ai‡bi hvbevnb e¨envi Ki‡Z হয়?  What is/ are the mode of transportation you used to reach to the health facility?  **DËi c‡o †kvbv‡eb bv|**  Do not read out the answers  **wR‡Ám Kiæbt AviI wKQy?**  ASK: Anything else?  **me DË‡ii †KvW e„ËvwqZ Kiæb|**  Circle code of all the answers | wiKkv / f¨vb Rickshaw/Rickshaw van | A |  |
|  |  | e¨vUvix wiKkv/UgUg/A‡UvwiKkv battery rickshaw/scooter | B |  |
|  |  | ‡bŠKv Boat | C |  |
|  |  | BwÄb PvwjZ ‡bŠKv Engine boat | D |  |
|  |  | evm / †U¤úy Bus/Tempo | E |  |
|  |  | Mvwo / gvB‡µvevm Car/Microbus | F |  |
|  |  | Ab¨vb¨ Other (wbw`©ó Kiæb) Specify ________________________ | Y |  |
| 204 | আপনার বাড়ি থেকে নিকটবর্তী স্বাস্থ্যকেন্দ্রে যাতায়াত করতে আপনার সাধারনত কত খরচ হয়? How much do you have to spend ususally to reach the health center and again come to home? | টাকা/ taka |  |  |
| 205 | evmv †_‡K †ei nevi ci নিকটবর্তী ¯^v¯’¨‡K‡›`ª †cŠQv‡bv ch©šÍ †gvU KZ mgq লাগে? What is the total time needed counting from when the patient started from home and to when the patient reached the referral health facility? | :  N›Uv : wgwbU |  |  |

**Section D: Pregnancy and ante natal care Mf© Ges Mf©Kvjxb †mev**

| **No.** | **Questions and filters** | | | | | | | **Responses** | | | | | | | | | | | | | **Code** | | | **Skip** |
| --- | --- | --- | --- | --- | --- | --- | --- | --- | --- | --- | --- | --- | --- | --- | --- | --- | --- | --- | --- | --- | --- | --- | --- | --- |
| 301 | **mv¶vZKviMÖnYKvixt**  **gwnjvi wK †Kvb Mf©** 1 RyjvB 2016 †_‡K 30 Ryb 2017 **Gi g‡a¨ †kl n‡q‡Q?**  Was there any pregnancy ended between **01 July, 2016 to 30^th^ May, 2017** for this woman? | | | | | | | **nu¨v** Yes | | | | | | | | | | | | | 1 | | |  |
|  |  |  |  |  |  |  |  | **bv** No | | | | | | | | | | | | | 2 | | | **🡪end** |
| 302 | **mv¶vZKviMÖnYKvixt Õ**শেষ **Mf© KZ gvm ¯’vqx n‡qwQj?Õ Ges mwVK †KvW e„ËvwqZ Kiæb|**  Ask duration of the last pregnancy of the woman? | | | | | | | 3 gvm ev Zvi Kg Pregnancy last less than 3 months | | | | | | | | | | | | | 1 | | | **🡪end** |
|  |  |  |  |  |  |  |  | 3 gv‡mi ‡ewk Pregnancy last more than 3 months | | | | | | | | | | | | | 2 | | |  |
| mv¶vZKviMÖnYKvixt 1 RyjvB 2016 †_‡K 30 Ryb 2017 Gi g‡a¨ DËi`vZvi me©‡kl M‡f©i Mf©Kvjxb hZœ m¤c‡K© wR‡Ám Ki‡Z n‡e, myZivs DËi`vZv‡K †mB Mf© m¤c‡K© fvj K‡i eywS‡q Zvici cÖkœ wR‡Ám Kiæb|  Interviewer: You have to collect information about the last pregnancy outcome (live-birth) during the period of 01 July 2016 to 30 June 2017. So, make sure that the respondent understand and identify the pregnancy then ask questions. | | | | | | | | | | | | | | | | | | | | | | | | |
| 303 | **GB Mf©Kvjxb mg‡q †gwW‡Kj †PKAvc Kwi‡qwQ‡jb wK?**  **mv¶vrKviMÖnbKvixt DËi`vZv‡K eywS‡q ejyb evwo‡Z A_ev Ab¨ †Kv_vI †h‡Kv‡bv ¯’v‡b †PKAvc Gi K_v Avcwb Rvb‡Z Pv‡”Qb|**  Did you see anyone for antenatal care for this pregnancy? | | | | | | | **nu¨v** Yes | | | | | | | | | | | | | 1 | | |  |
|  |  |  |  |  |  |  |  | **bv** No | | | | | | | | | | | | | 2 | | | 🡪308 |
| 304 | **Avcwb GB M‡f©i Rb¨ KZevi Mf©Kvjxb ‡mev (†PKAvc) Kwi‡q‡Qb?** How many times did you receive ANC during this pregnancy? | | | | | | | **evi** times | | | | | | | | | | | | | | | |  |
|  |  |  |  |  |  |  |  | **Rvwbbv/g‡b bvB** Don’t know | | | | | | | | | | | | | 97 | | |  |
| 305 | **Avcwb hLb GB M‡f©i Rb¨ Mf©Kvjxb ‡mev (†PKAvc) Kwi‡q‡Qb, ZLb Avcwb KZ gv‡mi Mf©eZx wQ‡jb?**  How many months pregnant were you when you received antenatal care for this pregnancy?  **mwVK gvm ej‡Z bv cvi‡j, Mf© gv‡mi N‡i** ‘99’ **wjLyb|**  Enter ’99’ if can’t remember months pregnant  (যতগুলো ANC মহিলা পেয়েছেন, কেবল তত সংখ্যক ANC এর তথ্য পূরণ করুন, বাকিগুলো ফাঁকা রাখুন) | | | | **ANC** visit **‡PKAvc** | | | | | | | | **nu¨v**  YES | | | **bv**  NO | | **Mf© gvm** Months pregnant | | | | | |  |
|  |  |  |  |  | ANC-1 | | | | | | | | 1 | | | 2 | |  | | | | | |  |
|  |  |  |  |  | ANC-2 | | | | | | | | 1 | | | 2 | |  | | | | | |  |
|  |  |  |  |  | ANC-3 | | | | | | | | 1 | | | 2 | |  | | | | | |  |
|  |  |  |  |  | ANC-4 | | | | | | | | 1 | | | 2 | |  | | | | | |  |
|  |  |  |  |  | ANC-5 | | | | | | | | 1 | | | 2 | |  | | | | | |  |
|  |  |  |  |  | ANC-6 | | | | | | | | 1 | | | 2 | |  | | | | | |  |
|  |  |  |  |  | ANC-7 | | | | | | | | 1 | | | 2 | |  | | | | | |  |
|  |  |  |  |  | ANC-8 | | | | | | | | 1 | | | 2 | |  | | | | | |  |
| 306 | **Avcwb Kv‡K †`wL‡qwQ‡jb?**  Whom did you see?  **cÖwZwU** ANC **Gi Rb¨ wRÁvmv Kiæb**  Ask for each ANC visit  **e¨w³ m¤ú©‡K wbwðZ †nvb Ges mwVK DË‡ii †KvW e„ËvwqZ Kiæb**  Probe to identify each type of person and record all mentioned. | **¯^v¯’¨‡mev`vbKvix** | | | | | **ANC 1** | | **ANC 2** | | **ANC 3** | | | **ANC 4** | **ANC 5** | | **ANC 6** | | **ANC 7** | | | **ANC 8** | |  |
|  |  | **¯^v¯’¨ ‡ckvRxwe (Health Personnel)** | | | | | | | | | | | | | | | | | | | | | |  |
|  |  | cvk Kiv Wv³vi Qualified doctor | | | | | 11 | | 11 | | 11 | | | 11 | 11 | | 11 | | 11 | | | 11 | |  |
|  |  | bvm©/avÎx/c¨viv‡gwWK Nurse/midwife/ Paramedic | | | | | 12 | | 12 | | 12 | | | 12 | 12 | | 12 | | 12 | | | 12 | |  |
|  |  | cwievi Kj¨vY cwi`wk©Kv FWV | | | | | 13 | | 13 | | 13 | | | 13 | 13 | | 13 | | 13 | | | 13 | |  |
|  |  | m¨vK‡gv SACMO | | | | | 14 | | 14 | | 14 | | | 14 | 14 | | 14 | | 14 | | | 14 | |  |
|  |  | wm,Gm,we,G CSBA | | | | | 15 | | 15 | | 15 | | | 15 | 15 | | 15 | | 15 | | | 15 | |  |
|  |  | wm,GBP, wm, wc CHCP | | | | | 16 | | 16 | | 16 | | | 16 | 16 | | 16 | | 16 | | | 16 | |  |
|  |  | ¯^v¯’¨ mnKvix HA | | | | | 17 | | 17 | | 17 | | | 17 | 17 | | 17 | | 17 | | | 17 | |  |
|  |  | cwievi Kj¨vb mnKvix FWA | | | | | 18 | | 18 | | 18 | | | 18 | 18 | | 18 | | 18 | | | 18 | |  |
|  |  | **Ab¨vb¨ ¯^v¯’¨ ‡mevcÖ`vbKvix (Other)** | | | | | | | | | | | | | | | | | | | | | |  |
|  |  | cÖwk¶Y cªvß wUweG (cÖwk¶Y cªvß abœx, PvDbx, `vB) TBA | | | | | 21 | | 21 | | 21 | | | 21 | 21 | | 21 | | 21 | | | 21 | |  |
|  |  | cÖwk¶Ynxb wUweG (abœx, PvDbx, `vB) UTBA(Dai/Dhorni/Chauni) | | | | | 22 | | 22 | | 22 | | | 22 | 22 | | 22 | | 22 | | | 22 | |  |
|  |  | mbvZb /cvk bv Kiv Wv³vi  Unqualified doctor | | | | | 23 | | 23 | | 23 | | | 23 | 23 | | 23 | | 23 | | | 23 | |  |
|  |  | Jla we‡µZv Drug seller | | | | | 24 | | 24 | | 24 | | | 24 | 24 | | 24 | | 24 | | | 24 | |  |
|  |  | GbwRI Kg©x NGO worker | | | | | 25 | | 25 | | 25 | | | 25 | 25 | | 25 | | 25 | | | 25 | |  |
|  |  | Ab¨vb¨ (wbw`©ó করুন) Other:_________ | | | | | 98 | | 98 | | 98 | | | 98 | 98 | | 98 | | 98 | | | 98 | |  |
|  |  | Rvwbbv/g‡b bvB Don’t know/Can’t remember | | | | | 99 | | 99 | | 99 | | | 99 | 99 | | 99 | | 99 | | | 99 | |  |
| 307 | **GB Mf©Kvjxb mg‡q ‡gwW‡Kj †PKAv†ci Rb¨ Avcwb †Kv_vq wM‡qwQ‡jb?**  Where did you receive antenatal care for this pregnancy?  **cÖwZwU** ANC **Gi Rb¨ wRÁvmv Kiæb**  Ask for each ANC visit  **‡cÖve K‡i wbwðZ †nvb wK ai‡bi Dr‡m wM‡qwQ‡jb Ges mwVK †KvW wjwce× Kiæb|**  Probe to identify each type of source.  **hw` wbwðZ n‡Z bv cv‡ib †h GUv miKvwi, bvwK cÖvB‡fU nvmcvZvj, wK¬wbK ev ¯^v¯’¨‡K›`ª Z‡e ¯’v‡bi bvg wj‡L ivLyb**  If unable to determine if public or private sector, write the name of the place:  ___________________  **¯’v‡bi bvg**(Name of place) | **Mf©Kvjxb †PKAv†ci ¯’vb** | | | | | **ANC 1** | | **ANC 2** | | **ANC 3** | | | **ANC 4** | **ANC**  **5** | | **ANC 6** | | **ANC 7** | | | **ANC 8** | |  |
|  |  | **evwo Home:** | | | | | | | | | | | | | | | | | | | | | |  |
|  |  | evwo‡Z Home | | | | | 11 | | 11 | | 11 | | | 11 | 11 | | 11 | | 11 | | | 11 | |  |
|  |  | **miKvix †mKUi Public sector:** | | | | | | | | | | | | | | | | | | | | | |  |
|  |  | †gwW‡Kj K‡jR nvmcvZvj Medical College | | | | | 21 | | 21 | | 21 | | | 21 | 21 | | 21 | | 21 | | | 21 | |  |
|  |  | we‡klvwqZ nvmcvZvj  Specialized hospital | | | | | 22 | | 22 | | 22 | | | 22 | 22 | | 22 | | 22 | | | 22 | |  |
|  |  | ‡Rjv nvmcvZvj District hospital | | | | | 23 | | 23 | | 23 | | | 23 | 23 | | 23 | | 23 | | | 23 | |  |
|  |  | gvZ…g½j †K›`ª (MCWC) | | | | | 24 | | 24 | | 24 | | | 24 | 24 | | 24 | | 24 | | | 24 | |  |
|  |  | Dc‡Rjv ¯^v¯’¨ Kg†cø·  Upazila Health Complex | | | | | 25 | | 25 | | 25 | | | 25 | 25 | | 25 | | 25 | | | 25 | |  |
|  |  | cwievi Kj¨vY †K›`ª  Family Welfare Centre (FWC) | | | | | 26 | | 26 | | 26 | | | 26 | 26 | | 26 | | 26 | | | 26 | |  |
|  |  | m¨v‡UjvBU wK¬wbK/BwcAvB †K›`ª Satellite clinic/EPI centre | | | | | 27 | | 27 | | 27 | | | 27 | 27 | | 27 | | 27 | | | 27 | |  |
|  |  | KwgDwbwU wK¬wbK Community clinic | | | | | 28 | | 28 | | 28 | | | 28 | 28 | | 28 | | 28 | | | 28 | |  |
|  |  | **Gb wR I †mKUi NGO sector:** | | | | | | | | | | | | | | | | | | | | | |  |
|  |  | Gb wR I ¯’vqx wK¬wbK NGO static clinic | | | | | 31 | | 31 | | 31 | | | 31 | 31 | | 31 | | 31 | | | 31 | |  |
|  |  | Gb wR I m¨v‡UjvBU wK¬wbK NGO satellite clinic | | | | | 32 | | 32 | | 32 | | | 32 | 32 | | 32 | | 32 | | | 32 | |  |
|  |  | **cÖvB‡fU †mKUi Private sector:** | | | | | | | | | | | | | | | | | | | | | |  |
|  |  | cÖvB‡fU nvmcvZvj / wK¬wbK Private hospital/Clinic | | | | | 41 | | 41 | | 41 | | | 41 | 41 | | 41 | | 41 | | | 41 | |  |
|  |  | cvk Kiv Wv³vi MBBS doctor (Qualified) | | | | | 42 | | 42 | | 42 | | | 42 | 42 | | 42 | | 42 | | | 42 | |  |
|  |  | AcÖwkw¶Z Wv³vi (†KvqvK/ cj­x wPwKrmK/ †nvwgIc¨v_) Quack/ Village doctor /Aiurved /Homeopath | | | | | 43 | | 43 | | 43 | | | 43 | 43 | | 43 | | 43 | | | 43 | |  |
|  |  | dv‡g©mx Pharmacy | | | | | 44 | | 44 | | 44 | | | 44 | 44 | | 44 | | 44 | | | 44 | |  |
|  |  | cÖvB‡fU †gwW‡Kj K‡jR nvmcvZvj Private Medical College | | | | | 46 | | 46 | | 46 | | | 46 | 46 | | 46 | | 46 | | | 46 | |  |
|  |  | Ab¨vb¨ (wbw`©ó করুন) Other:  ________________________ | | | | | 98 | | 98 | | 98 | | | 98 | 98 | | 98 | | 98 | | | 98 | |  |
|  |  | Rvwbbv/g‡b bvB DK/Can’t remember | | | | | 99 | | 99 | | 99 | | | 99 | 99 | | 99 | | 99 | | | 99 | |  |
| 308 | **Avcwb wK GB Mf©Kvjxb mg‡q (bvg †c‡U _vKvKvjxb) beRvZ‡Ki abyósKvi cÖwZ‡iv‡a wU,wU Bb‡RKkb wb‡q‡Qb?** During this pregnancy, were you given an injection in the arm to prevent the baby from getting tetanus, that is, convulsions after birth? | | | | | | | | | | | **nu¨v** Yes | | | | | | | | | | | 1 |  |
|  |  |  |  |  |  |  |  |  |  |  |  | **bv** No | | | | | | | | | | | 2 | 🡪311 |
|  |  |  |  |  |  |  |  |  |  |  |  | **Rvwbbv/g‡b bvB** Don’t know/Can’t remember | | | | | | | | | | | 97 | 🡪311 |
| 309 | **GB Mf©Kvjxb mg‡q Avcwb KZevi wU,wU Bb‡RKkb wb‡q‡Qb?** During this pregnancy, how many times did you get a tetanus injection (TT)? | | | | | | | | | **evi** times | | | | | | | | | | | | | |  |
|  |  |  |  |  |  |  |  |  |  | **Rvwbbv/g‡b bvB** Don’t know | | | | | | | | | | | 97 | | |  |
| 310 | **mv¶vZKviMÖnYKvixt cÖkœ** 309 **†`Lyb Ges mwVK †KvW e„ËvwqZ Kiæb|** Check Q-309 and circle appropriate code | | | | | | | | | **`yB ev Z‡ZvwaK** 2 or more times | | | | | | | | | | | 1 | | | 🡪314 |
|  |  |  |  |  |  |  |  |  |  | **Ab¨vb¨** Other **(একবার/ একবারও না)** | | | | | | | | | | | 2 | | |  |
| 311 | **GB M‡f©i c~‡e© Avcwb KLbI wU,wU Bb‡RKkb wb‡q‡Qb?** At any time before this pregnancy, did you receive any tetanus injections (TT)? | | | | | | | | | **nu¨v** Yes | | | | | | | | | | | 1 | | |  |
|  |  |  |  |  |  |  |  |  |  | **bv** No | | | | | | | | | | | 2 | | | 🡪314 |
|  |  |  |  |  |  |  |  |  |  | **Rvwbbv/g‡b bvB** Don’t know/Can’t remember | | | | | | | | | | | 97 | | | 🡪314 |
| 312 | **GB M‡f©i c~‡e© Avcwb KZevi wU,wU Bb‡RKkb wb‡q‡Qb?** Before this pregnancy, how many times did you receive a tetanus injection?  **7 evi ev Zvi †ekx n‡j** ‘07’ **wjLyb|** If 7 or more times, record ‘07’. | | | | | | | | | **evi** times | | | | | | | | | | | | | |  |
|  |  |  |  |  |  |  |  |  |  | **Rvwbbv/g‡b bvB** Don’t know | | | | | | | | | | 97 | | | |  |
| 313 | **GB M‡f©i KZ eQi Av‡M Avcwb †kl wU,wU wUKvwU wb‡q‡Qb?** How many years ago did you receive the last tetanus injection before this pregnancy?  **এক বছরের কম হলে** “মাস” এর **ঘর পূরণ করুন ও** “1” কোড করুন । এক বছর অথবা এর বেশি হলে “বছর” এর ঘরে পূর্ণ বছর লিখুন ও “2”কোড করুন। | | | | | | | | | **মাস আগে** Months ago | | | | | | | | | | 1 | | | |  |
|  |  |  |  |  |  |  |  |  |  | **eQi Av‡M** Years ago | | | | | | | | | | 2 | | | |  |
|  |  |  |  |  |  |  |  |  |  | মনে নাই Can’t remember | | | | | | | | | | 97 | | | |  |
| 314 | **GB Mf©Kvjxb mg‡q Avcwb (kix‡i i³ nIqvi Rb¨) Avqib U¨v‡e‡jU ev Avqib wmivc †L‡qwQ‡jb wK?** Did you take iron tablets or syrup during this pregnancy? | | | | | | | | | **nu¨v** Yes | | | | | | | | | | 1 | | | |  |
|  |  |  |  |  |  |  |  |  |  | **bv** No | | | | | | | | | | 2 | | | | 🡪318 |
|  |  |  |  |  |  |  |  |  |  | **Rvwbbv/g‡b bvB** Don’t know/Can’t remember | | | | | | | | | | 97 | | | | 🡪318 |
| 315 | **GB Mf©Kvjxb mg‡q** Avcwb †gvU KZ w`b Avqib-d‡jU U¨ve‡jU/ wmivc খেয়েছেন? How many days did you take the iron tablets/syrup during your this pregnancy? | | | | | | | | | w`b Days | | | | | | | | | | | | | |  |
|  |  |  |  |  |  |  |  |  |  | Rvwbbv/g‡b bvB Don’t know | | | | | | | | | | | 999 | | |  |
| 316 | **GB Mf©Kvjxb mg‡q** †gvU KZ¸‡jv Avqib-d‡jU U¨ve‡jU A_ev Avqib wmivc **†c‡qwQ‡jb A_ev কিনেছেন**? How many tablets/bottles did you buy or receive during your this pregnancy? | | | | | | | | | msL¨K U¨ve‡jU number of tablets | | | | | | | | | | | | | |  |
|  |  |  |  |  |  |  |  |  |  | msL¨K †evZj number of bottles | | | | | | | | | | | | | |  |
|  |  |  |  |  |  |  |  |  |  | একটাও পাইনি Received none | | | | | | | | | | | 998 | | |  |
|  |  |  |  |  |  |  |  |  |  | Rvwbbv/g‡b bvB Don’t know | | | | | | | | | | | 999 | | |  |
| 317 | hLb Avcwb Mf©eZx wQ‡jb, ZLb Avcwb †Kv_v †_‡K Avqib-d‡jU U¨ve‡jU **†c‡qwQ‡jb A_ev কিনেছেন**?  [gwnjv‡K wR‡Ám Kiæb] AviI wKQz?  [gwnjvi wb‡R †_‡K †`qv me¸‡jv DËiB e„ËvwqZ Kiæb | DËi¸‡jv c‡o ïbv‡eb bv| GKvwaK DËi n‡Z cv‡i|]  Where did you get this calcium tablets or syrup? [Do not read out the answers. Ask: Anything else? Circle all the answers] | | **evox (Home)** | | | | | | | | | | | | | | | | | |  | | |  |
|  |  |  | evox‡Z, miKvix ¯^v¯’¨Kgx©i KvQ †_‡K (Duing GoB health worker’s home visit) | | | | | | | | | | | | | | | | | | A | | |  |
|  |  |  | evox‡Z, ‡emiKvix / GbwRI ¯^v¯’¨Kgx©i KvQ †_‡K (Duing Private/NGO health worker’s home visit ) | | | | | | | | | | | | | | | | | | B | | |  |
|  |  |  | evox‡Z, AvZ¥x‡qi KvQ †_‡K (From realtive) | | | | | | | | | | | | | | | | | | C | | |  |
|  |  |  | **miKvix ¯^v¯’¨ †K›`ª (Govt Health center)** | | | | | | | | | | | | | | | | | |  | | |  |
|  |  |  | †gwW‡Kj K‡jR nvmcvZvj Medical College | | | | | | | | | | | | | | | | | | D | | |  |
|  |  |  | we‡klvwqZ nvmcvZvj Specialized hospital | | | | | | | | | | | | | | | | | | E | | |  |
|  |  |  | ‡Rjv nvmcvZvj District hospital | | | | | | | | | | | | | | | | | | F | | |  |
|  |  |  | gvZ…g½j †K›`ª Maternal & Child Welfare Centre (MCWC) | | | | | | | | | | | | | | | | | | G | | |  |
|  |  |  | Dc‡Rjv ¯^v¯’¨ Kg†cø· Upazila Health Complex | | | | | | | | | | | | | | | | | | H | | |  |
|  |  |  | cwievi Kj¨vY †K›`ª Family Welfare Centre (FWC) | | | | | | | | | | | | | | | | | | I | | |  |
|  |  |  | m¨v‡UjvBU wK¬wbK/BwcAvB †K›`ª Satellite clinic/EPI centre | | | | | | | | | | | | | | | | | | J | | |  |
|  |  |  | KwgDwbwU wK¬wbK Community clinic | | | | | | | | | | | | | | | | | | K | | |  |
|  |  |  | Ab¨vb¨(wbw`©ó Kiæb) Other: | | | | | | | | | | | | | | | | | | L | | |  |
|  |  |  | **Gb wR I †mKUi NGO sector:** | | | | | | | | | | | | | | | | | |  | | |  |
|  |  |  | GbwRI nvmcvZvj (NGO hospital) | | | | | | | | | | | | | | | | | | M | | |  |
|  |  |  | Gb wR I ¯’vqx wK¬wbK NGO static clinic | | | | | | | | | | | | | | | | | | N | | |  |
|  |  |  | Gb wR I m¨v‡UjvBU wK¬wbK NGO satellite clinic | | | | | | | | | | | | | | | | | | O | | |  |
|  |  |  | Ab¨vb¨(wbw`©ó Kiæb) Other: | | | | | | | | | | | | | | | | | | P | | |  |
|  |  |  | **cÖvB‡fU †mKUi Private sector:** | | | | | | | | | | | | | | | | | |  | | |  |
|  |  |  | cÖvB‡fU nvmcvZvj / wK¬wbK Private hospital/Clinic | | | | | | | | | | | | | | | | | | Q | | |  |
|  |  |  | cvk Kiv Wv³v‡ii †P¤^vi MBBS doctor (Qualified) | | | | | | | | | | | | | | | | | | R | | |  |
|  |  |  | MÖvg Wv³v‡ii †P¤^vi (Village doctor’s chamber) | | | | | | | | | | | | | | | | | | S | | |  |
|  |  |  | mbvZb/AcÖwkw¶Z Wv³v‡ii †P¤^vi Traditional doctor | | | | | | | | | | | | | | | | | | T | | |  |
|  |  |  | dv‡g©mx/Ily‡ai †`vKvb Pharmacy | | | | | | | | | | | | | | | | | | U | | |  |
|  |  |  | cÖvB‡fU †gwW‡Kj K‡jR nvmcvZvjPrivate Medical College | | | | | | | | | | | | | | | | | | V | | |  |
|  |  |  | Ab¨vb¨(wbw`©ó Kiæb) Other: | | | | | | | | | | | | | | | | | | Y | | |  |
| 318 | **GB Mf©Kvjxb mg‡q Avcwb K¨vjwmqvg U¨v‡e‡jU ev wmivc †L‡qwQ‡jb wK?** Did you take calcium tablets or syrup during this pregnancy? | | | **nu¨v** Yes | | | | | | | | | | | | | | | | | 1 | | |  |
|  |  |  |  | **bv** No | | | | | | | | | | | | | | | | | 2 | | | 🡪322 |
|  |  |  |  | **Rvwbbv/g‡b bvB** Don’t know/Can’t remember | | | | | | | | | | | | | | | | | 97 | | | 🡪322 |
| 319 | **GB Mf©Kvjxb mg‡q** Avcwb †gvU KZ w`b **K¨vjwmqvg U¨v‡e‡jU ev wmivc** খেয়েছেন? How many days did you take the calcium tablets/syrup during your this pregnancy? | | | w`b Days | | | | | | | | | | | | | | | | | | | |  |
|  |  |  |  | Rvwbbv/g‡b bvB Don’t know | | | | | | | | | | | | | | | | | 999 | | |  |
| 320 | **GB Mf©Kvjxb mg‡q** (পুরোটা সময়) †gvU KZ¸‡jv **K¨vjwmqvg U¨v‡e‡jU ev wmivc** **†c‡qwQ‡jb A_ev কিনেছেন**? How many calcium tablets/bottles did you buy or receive during your this pregnancy? | | | msL¨K U¨ve‡jU number of tablets | | | | | | | | | | | | | | | | | | | |  |
|  |  |  |  | msL¨K †evZj number of bottles | | | | | | | | | | | | | | | | | | | |  |
|  |  |  |  | একটাও পাইনি Received none | | | | | | | | | | | | | | | | | 998 | | |  |
|  |  |  |  | Rvwbbv/g‡b bvB Don’t know | | | | | | | | | | | | | | | | | 999 | | |  |
| 321 | hLb Avcwb Mf©eZx wQ‡jb, ZLb Avcwb †Kv_v †_‡K **K¨vjwmqvg U¨v‡e‡jU ev wmivc †c‡qwQ‡jb A_ev কিনেছেন**?  [gwnjv‡K wR‡Ám Kiæb] AviI wKQz?  [gwnjvi wb‡R †_‡K †`qv me¸‡jv DËiB e„ËvwqZ Kiæb | DËi¸‡jv c‡o ïbv‡eb bv| GKvwaK DËi n‡Z cv‡i|]  Where did you get this calcium tablets or syrup? [Do not read out the answers. Ask: Anything else? Circle all the answers] | | **evox (Home)** | | | | | | | | | | | | | | | | | |  | | |  |
|  |  |  | evox‡Z, miKvix ¯^v¯’¨Kgx©i KvQ †_‡K (Duing GoB health worker’s home visit) | | | | | | | | | | | | | | | | | | A | | |  |
|  |  |  | evox‡Z, ‡emiKvix / GbwRI ¯^v¯’¨Kgx©i KvQ †_‡K (Duing Private/NGO health worker’s home visit ) | | | | | | | | | | | | | | | | | | B | | |  |
|  |  |  | evox‡Z, AvZ¥x‡qi KvQ †_‡K (From realtive) | | | | | | | | | | | | | | | | | | C | | |  |
|  |  |  | **miKvix ¯^v¯’¨ †K›`ª (Govt Health center)** | | | | | | | | | | | | | | | | | |  | | |  |
|  |  |  | †gwW‡Kj K‡jR nvmcvZvj Medical College | | | | | | | | | | | | | | | | | | D | | |  |
|  |  |  | we‡klvwqZ nvmcvZvj Specialized hospital | | | | | | | | | | | | | | | | | | E | | |  |
|  |  |  | ‡Rjv nvmcvZvj District hospital | | | | | | | | | | | | | | | | | | F | | |  |
|  |  |  | gvZ…g½j †K›`ª Maternal & Child Welfare Centre (MCWC) | | | | | | | | | | | | | | | | | | G | | |  |
|  |  |  | Dc‡Rjv ¯^v¯’¨ Kg†cø· Upazila Health Complex | | | | | | | | | | | | | | | | | | H | | |  |
|  |  |  | cwievi Kj¨vY †K›`ª Family Welfare Centre (FWC) | | | | | | | | | | | | | | | | | | I | | |  |
|  |  |  | m¨v‡UjvBU wK¬wbK/BwcAvB †K›`ª Satellite clinic/EPI centre | | | | | | | | | | | | | | | | | | J | | |  |
|  |  |  | KwgDwbwU wK¬wbK Community clinic | | | | | | | | | | | | | | | | | | K | | |  |
|  |  |  | Ab¨vb¨(wbw`©ó Kiæb) Other:_____________________________ | | | | | | | | | | | | | | | | | | L | | |  |
|  |  |  | **Gb wR I †mKUi NGO sector:** | | | | | | | | | | | | | | | | | |  | | |  |
|  |  |  | GbwRI nvmcvZvj (NGO hospital) | | | | | | | | | | | | | | | | | | M | | |  |
|  |  |  | Gb wR I ¯’vqx wK¬wbK NGO static clinic | | | | | | | | | | | | | | | | | | N | | |  |
|  |  |  | Gb wR I m¨v‡UjvBU wK¬wbK NGO satellite clinic | | | | | | | | | | | | | | | | | | O | | |  |
|  |  |  | Ab¨vb¨(wbw`©ó Kiæb) Other: | | | | | | | | | | | | | | | | | | P | | |  |
|  |  |  | **cÖvB‡fU †mKUi Private sector:** | | | | | | | | | | | | | | | | | |  | | |  |
|  |  |  | cÖvB‡fU nvmcvZvj / wK¬wbK Private hospital/Clinic | | | | | | | | | | | | | | | | | | Q | | |  |
|  |  |  | cvk Kiv Wv³v‡ii †P¤^vi MBBS doctor (Qualified) | | | | | | | | | | | | | | | | | | R | | |  |
|  |  |  | MÖvg Wv³v‡ii †P¤^vi (Village doctor’s chamber) | | | | | | | | | | | | | | | | | | S | | |  |
|  |  |  | mbvZb/AcÖwkw¶Z Wv³v‡ii †P¤^vi Traditional doctor | | | | | | | | | | | | | | | | | | T | | |  |
|  |  |  | dv‡g©mx/Ily‡ai †`vKvb Pharmacy | | | | | | | | | | | | | | | | | | U | | |  |
|  |  |  | cÖvB‡fU †gwW‡Kj K‡jR nvmcvZvjPrivate Medical College | | | | | | | | | | | | | | | | | | V | | |  |
|  |  |  | Ab¨vb¨(wbw`©ó Kiæb) Other:________________________________ | | | | | | | | | | | | | | | | | | Y | | |  |
| 322 | **GB Mf©Kvjxb mg‡q Avcbvi †Kvb mgm¨v ev RwUjZv n‡qwQj wK hvi Rb¨ Wv³vix wPwKrmvi cÖ‡qvRb wQj?** During the pregnancy with (NAME), did you develop any problem/complication that required medical treatment? | | | | | **nu¨v** Yes | | | | | | | | | | | | | | | 1 | | |  |
|  |  |  |  |  |  | **bv** No | | | | | | | | | | | | | | | 2 | | | 🡪400 |
|  |  |  |  |  |  | **Rvwbbv/g‡b bvB** Don’t know/Can’t remember | | | | | | | | | | | | | | | 97 | | | 🡪400 |
| 323 | **Avcbvi wK ai‡Yi mgm¨v ev RwUjZv n‡qwQj?**  Please tell me what was that problem or complication?  **DËi c‡o †kvbv‡eb bv|**  Do not read out the answers  **wR‡Ám Kiæbt AviI wKQy?**  ASK: Anything else?  **me DË‡ii †KvW e„ËvwqZ Kiæb|**  Circle code of all the answers | | Zxeª gv_v e¨v_v Severe Headache | | | | | | | | | | | | | | | | | | A | | |  |
|  |  |  | ‡Pv‡L Svcmv †`Lv Blurred Vision | | | | | | | | | | | | | | | | | | B | | |  |
|  |  |  | M‡f©i ev”Pvi bovPov K‡g hvIqv Fetal movement reduced | | | | | | | | | | | | | | | | | | C | | |  |
|  |  |  | M‡f©i ev”Pvi bovPov eÜ nIqv Fetal movement absent | | | | | | | | | | | | | | | | | | D | | |  |
|  |  |  | gyLgÛ‡j cvwb Avmv/dz‡j hvIqv Oedema of the face/swelling | | | | | | | | | | | | | | | | | | E | | |  |
|  |  |  | nv‡Z cvwb Avmv/dz‡j hvIqv Oedema of the hands/swelling | | | | | | | | | | | | | | | | | | F | | |  |
|  |  |  | wLuPzbx/wdU Convulsions/fits | | | | | | | | | | | | | | | | | | G | | |  |
|  |  |  | ev”Pv nIqvi iv¯Zvq AwZwi³ i³mªve Excessive Vaginal Bleeding | | | | | | | | | | | | | | | | | | H | | |  |
|  |  |  | Zj‡c‡U Zxeª e¨_v Severe abdominal pain | | | | | | | | | | | | | | | | | | I | | |  |
|  |  |  | cv‡q cvwb Avmv Oedema of the legs | | | | | | | | | | | | | | | | | | J | | |  |
|  |  |  | Zxeª R¡i Fever | | | | | | | | | | | | | | | | | | K | | |  |
|  |  |  | mgq c‚Y© nIqvi Av‡M cvwb fv½v Premature rupture of membrane | | | | | | | | | | | | | | | | | | L | | |  |
|  |  |  | A‡PZb nIqv/Ávb nvwi‡q †djv Loss of consciousness | | | | | | | | | | | | | | | | | | M | | |  |
|  |  |  | Kó K‡i k¦vm †bqv Difficulty breathing | | | | | | | | | | | | | | | | | | N | | |  |
|  |  |  | cÖPÛ `ye©jZv Severe weakness | | | | | | | | | | | | | | | | | | O | | |  |
|  |  |  | AwZwi³ ewg Excessive vomiting | | | | | | | | | | | | | | | | | | P | | |  |
|  |  |  | AwZwi³ mv`v mªve Excessive whitish vaginal discharge | | | | | | | | | | | | | | | | | | Q | | |  |
|  |  |  | Ab¨vb¨ (wbw`©ó Kiæb) Others_________________________ | | | | | | | | | | | | | | | | | | X | | |  |
|  |  |  | Rvwbbv/wKQyB D‡j­L K‡ibwb Don’t know/None mentioned | | | | | | | | | | | | | | | | | | Y | | |  |
| 324 | **GB mgm¨v/Amyweav/RwUjZvi Rb¨ Avcwb †Kvb wPwKrmv Kwi‡q‡Qb wK?**  Did you seek any sort of treatment for this problem/complication? | | | | | | **nu¨v** Yes | | | | | | | | | | | | | | 1 | | |  |
|  |  |  |  |  |  |  | **bv** No | | | | | | | | | | | | | | 2 | | | 🡪400 |
|  |  |  |  |  |  |  | **Rvwbbv/g‡b bvB** Don’t know/Can’t remember | | | | | | | | | | | | | | 97 | | | 🡪400 |
| 325 | **Avcwb Kvi Kv‡Q wPwKrmv Kwi‡q‡Qb?**  From whom you received treatment for this problem/complication?  **wR‡Ám Kiæbt Avi ‡KD?** Anyone else?  **e¨w³ m¤ú©‡K wbwðZ †nvb Ges mwVK DË‡ii †KvW e„ËvwqZ Kiæb**  Probe to identify each type of person and check all mentioned. | | cvk Kiv Wv³vi MBBS doctor (Qualified) | | | | | | | | | | | | | | | | | | A | | |  |
|  |  |  | bvm©/ avÎx /c¨viv‡gwWK Nurse/Midwife/Paramedic | | | | | | | | | | | | | | | | | | B | | |  |
|  |  |  | cwievi Kj¨vY cwi`wk©Kv Family Welfare Visitor (FWV | | | | | | | | | | | | | | | | | | C | | |  |
|  |  |  | KwgDwbwU wfwËK `¶ avÎx (CSBA) | | | | | | | | | | | | | | | | | | D | | |  |
|  |  |  | KwgDwbwU wK¬wbK wfwËK ¯^v¯’¨ †mev cÖ`vbKvix (CHCP) | | | | | | | | | | | | | | | | | | E | | |  |
|  |  |  | wPwKrmv mnKvix / DcmnKvix KwgDwbwU wPwKrmv Kg©KZ©v Medical Assistant / SACMO | | | | | | | | | | | | | | | | | | F | | |  |
|  |  |  | ¯^v¯’¨ mnKvix Health Assistant (HA) | | | | | | | | | | | | | | | | | | G | | |  |
|  |  |  | cwievi Kj¨vY mnKvix Family Welfare Assistant (FWA) | | | | | | | | | | | | | | | | | | H | | |  |
|  |  |  | cÖwk¶YcÖvß wUweG/`vB Trained TBA | | | | | | | | | | | | | | | | | | I | | |  |
|  |  |  | AcÖwkw¶Z wUweG/`vB Untrained TBA | | | | | | | | | | | | | | | | | | J | | |  |
|  |  |  | AcÖwkw¶Z Wv³vi (MÖvg Wv³vi/ ‡nvwgIc¨v_/ KweivR) Unqualified doctor | | | | | | | | | | | | | | | | | | K | | |  |
|  |  |  | Gb wR I ¯^v¯’¨Kg©x NGO worker | | | | | | | | | | | | | | | | | | L | | |  |
|  |  |  | cwiev‡ii m`m¨/AvZœxq/cÖwZ‡ekx Family member/ Relative/ Neighbour | | | | | | | | | | | | | | | | | | M | | |  |
|  |  |  | **dv‡g©wm** Pharmacy | | | | | | | | | | | | | | | | | | N | | |  |
|  |  |  | **Ab¨vb¨** (wbw`©ó Kiæb) Other__________________________________ | | | | | | | | | | | | | | | | | | X | | |  |
|  |  |  | Rvwbbv/g‡b bvB Don’t know | | | | | | | | | | | | | | | | | | Y | | |  |
| 326 | **GB wPwKrmv Avcwb †Kv_vq Kwi‡q‡Qb?**  From where did you receive care for this problem/complication?  **wR‡Ám Kiæbt Avi ‡Kv_vI?** Anywhere else?  **¯’vb m¤ú©‡K wbwðZ †nvb Ges mwVK DË‡ii †KvW e„ËvwqZ Kiæb**  Probe to identify each type of source.  **wbw`©ó †Kv‡Wi evwn‡ii †Kvb ¯’vb n‡j, bx‡P ¯’v‡bi bvg wjLyb**  If unable to determine the place, write the name of the place below:  ____________________  ¯’v‡bi bvg (Name of place) | | evwo‡Z Home | | | | | | | | | | | | | | | | | | A | | |  |
|  |  |  | **miKvix †mKUi Public sector:** | | | | | | | | | | | | | | | | | |  | | |  |
|  |  |  | †gwW‡Kj K‡jR nvmcvZvj Medical College | | | | | | | | | | | | | | | | | | B | | |  |
|  |  |  | we‡klvwqZ nvmcvZvj Specialized hospital | | | | | | | | | | | | | | | | | | C | | |  |
|  |  |  | ‡Rjv nvmcvZvj District hospital | | | | | | | | | | | | | | | | | | D | | |  |
|  |  |  | gvZ…g½j †K›`ª Maternal & Child Welfare Centre (MCWC) | | | | | | | | | | | | | | | | | | E | | |  |
|  |  |  | Dc‡Rjv ¯^v¯’¨ Kg†cø· Upazila Health Complex | | | | | | | | | | | | | | | | | | F | | |  |
|  |  |  | cwievi Kj¨vY †K›`ª Family Welfare Centre (FWC) | | | | | | | | | | | | | | | | | | G | | |  |
|  |  |  | m¨v‡UjvBU wK¬wbK/BwcAvB †K›`ª Satellite clinic/EPI centre | | | | | | | | | | | | | | | | | | H | | |  |
|  |  |  | KwgDwbwU wK¬wbK Community clinic | | | | | | | | | | | | | | | | | | I | | |  |
|  |  |  | **Gb wR I †mKUi NGO sector:** | | | | | | | | | | | | | | | | | |  | | |  |
|  |  |  | Gb wR I ¯’vqx wK¬wbK NGO static clinic | | | | | | | | | | | | | | | | | | J | | |  |
|  |  |  | Gb wR I m¨v‡UjvBU wK¬wbK NGO satellite clinic | | | | | | | | | | | | | | | | | | K | | |  |
|  |  |  | **cÖvB‡fU †mKUi Private sector:** | | | | | | | | | | | | | | | | | |  | | |  |
|  |  |  | cÖvB‡fU nvmcvZvj / wK¬wbK Private hospital/Clinic | | | | | | | | | | | | | | | | | | L | | |  |
|  |  |  | cvk Kiv Wv³vi MBBS doctor (Qualified) | | | | | | | | | | | | | | | | | | M | | |  |
|  |  |  | AcÖwkw¶Z Wv³vi (†KvqvK/ cj­x wPwKrmK/ †nvwgIc¨v_) Quack/ Village doctor /Aiurved /Homeopath | | | | | | | | | | | | | | | | | | N | | |  |
|  |  |  | dv‡g©mx Pharmacy | | | | | | | | | | | | | | | | | | O | | |  |
|  |  |  | cÖvB‡fU †gwW‡Kj K‡jR nvmcvZvj Private Medical College | | | | | | | | | | | | | | | | | | P | | |  |
|  |  |  | Ab¨vb¨ (wbw`©ó করুন) Other:_________________________ | | | | | | | | | | | | | | | | | | Q | | |  |
|  |  |  | Ab¨vb¨ Other sources: | | | | | | | | | | | | | | | | | |  | | |  |
|  |  |  | **‡`vKvb** Shop | | | | | | | | | | | | | | | | | | R | | |  |
|  |  |  | **eÜz/AvZ¥xq** Friends/Relatives | | | | | | | | | | | | | | | | | | S | | |  |
|  |  |  | **wUweG / `vB** TBA | | | | | | | | | | | | | | | | | | T | | |  |
|  |  |  | **Ab¨vb¨** (wbw`©ó Kiæb) Other | | | | | | | | | | | | | | | | | | X | | |  |

**Section E: Delivery cªme**

| **No.** | **Questions and filters** | **Responses** | | **Code** | **Skip** |
| --- | --- | --- | --- | --- | --- |
| 400 | **mv¶vZKvi MÖnbKvixt gwnjvi me©‡kl M‡f©i djvdj m¤ú‡K© mwVK †KvW e„ËvwqZ Kiæb|** What was the outcome of her last pregnancy? | **RxweZ Rb¥** Live birth | | 1 |  |
|  |  | **g„Z Rb¥** Still birth | | 2 |  |
| GLb Avwg Avcbvi 1 RyjvB 2016 †_‡K 30 Ryb 2017 Gi g‡a¨ nIqv me©‡kl †Wwjfvwi m¤c‡K© wKQy cÖkœ wR‡Ám Kie|  Now I shall ask few questions about delivery of (NAME) born between 01 July 2016 to 30 June 2017. | | | | |  |
| 401 | **†Wwjfvwi wU †Kv_vq n‡qwQj?**  Where did the birth /delvery take place? | **evwo** Home | | 11 |  |
|  |  | **miKvix ¯^v¯’¨ †K›`ª Public sector:** | |  |  |
|  |  | **KwgDwbwU wK¬wbK** Community clinic | | 21 |  |
|  |  | **BDwbqb ¯^v¯’¨ I cwievi Kj¨vY †K›`ª** UH&FWC | | 22 |  |
|  |  | **Dc‡Rjv ¯^v¯’¨ Kg‡cø·** Upazila Health Complex (UHC) | | 23 |  |
|  |  | **†Rjv nvmcvZvj** District hospital | | 24 |  |
|  |  | **†gwW‡Kj K‡jR nvmcvZvj** Medical College Hospital | | 25 |  |
|  |  | **gvZ…g½j †K›`ª** MCWC / Maternity centre | | 26 |  |
|  |  | **Ab¨vb¨ miKvix ¯^v¯’¨ †K›`ª** Other public health centre  _______________________________ | | 27 |  |
|  |  | **Gb,wR,I/cÖvB‡fU ¯^v¯’¨ †K›`ª NGO/Private health centre** | |  |  |
|  |  | **Gb wR I wK¬wbK/nvmcvZvj** NGO clinic/hospital | | 31 |  |
|  |  | **cÖvB‡fU nvmcvZvj/wK¬wbK** Private hospital/clinic | | 32 |  |
|  |  | **¯^v¯’¨ †K›`ª Qvov Ab¨ †Kv_vI** (wbw`©ó করুন)  Others | | 33 |  |
| 402 | **†Wwjfvwi †K Kwi‡qwQ‡jb?**  Who assisted with the delivery of (NAME)?  **mv¶vZKviMÖnYKvixt †Wwjfvwi‡Z cÖavbZ whwb mvnvh¨ K‡iwQ‡jb A_¨©vr hvi f‚wgKv me‡P‡q †ewk wQj Zvi †KvW e„ËvwqZ** করুন  Interviewer: Ask and record the designation of the person who mainly responsible or mostly involved in assisting the delivery | ¯^v¯’¨ †ckvRxwet Health personnel: | |  |  |
|  |  | **cvk Kiv Wv³vi** MBBS doctor (Qualified) | | 11 |  |
|  |  | bvm©/ avÎx /c¨viv‡gwWK Nurse/Midwife/Paramedic | | 12 |  |
|  |  | cwievi Kj¨vY cwi`wk©Kv Family Welfare Visitor (FWV) | | 13 |  |
|  |  | wPwKrmv mnKvix / DcmnKvix KwgDwbwU wPwKrmv Kg©KZ©v Medical Assistant / SACMO | | 14 |  |
|  |  | KwgDwbwU wfwËK `¶ avÎx (CSBA) | | 15 |  |
|  |  | KwgDwbwU wK¬wbK wfwËK ¯^v¯’¨ †mev cÖ`vbKvix (CHCP) | | 16 |  |
|  |  | ¯^v¯’¨ mnKvix Health Assistant (HA) | | 17 |  |
|  |  | cwievi Kj¨vY mnKvix Family Welfare Assistant (FWA) | | 18 |  |
|  |  | Ab¨vb¨ e¨w³t Other person: | |  |  |
|  |  | **cÖwk¶YcÖvß wUweG** Trained TBA | | 21 |  |
|  |  | **AcÖwkw¶Z wUweG** Untrained TBA | | 22 |  |
|  |  | **AcÖwkw¶Z Wv³vi (MÖvg Wv³vi/ cjøx wPwKrmK/‡nvwgIc¨v_/ KweivR)** Unqualified doctor | | 23 |  |
|  |  | **Gb wR I ¯^v¯’¨Kg©x** NGO worker | | 24 |  |
|  |  | **cwiev‡ii m`m¨/AvZœxq** Family member / Relative | | 25 |  |
|  |  | **cÖwZ‡ekx/eÜz** Neighbour / Friend | | 26 |  |
|  |  | **Ab¨vb¨** (wbw`©ó Kiæb) Other: | | 27 |  |
|  |  | **Rvwbbv/g‡b bvB** Don’t know/Can’t remember | | 97 |  |
| 403 | **cÖm‡ei aib wK wQj?**  What was the mode of delivery? | **¯^vfvweK cÖme** Normal delivery | | 1 |  |
|  |  | **di‡mcm †Wwjfvwi** Forceps delivery | | 2 |  |
|  |  | **f¨vKzqvg/ †fb‡Uvm** Vacuum/Ventouse | | 3 |  |
|  |  | **wmRvwiqvb †mKkb** Caesarean section | | 4 |  |
| 404 | **mv¶vZKviMÖnbKvixt cÖkœ** 401 **‡`Lyb Ges †WwjfvwiwU †Kv_vq n‡q‡Q Zv e„ËvwqZ Kiæb|**  Check question 401 and circle code of place of delivery. | **evwo‡Z** At Home (Code 11) | | 1 |  |
|  |  | **¯^v¯’¨‡K‡›`ª** At Health Facility (code 21-33) | | 2 | 🡪408 |
|  |  | **Ab¨vb¨** Any other place (Code 96) | | 3 |  |
| 405 | **†Wwjfvwi‡Z whwb mvnvh¨ K‡iwQ‡jb, †Wwjfvwii c~‡e© wZwb Zvi nvZ mvevb w`‡q ay‡qwQ‡jb wK?**  Did the person who assisted with the delivery of (NAME) wash hands with soap prior to delivery? | **nu¨v** Yes | | 1 |  |
|  |  | **bv** No | | 2 |  |
|  |  | **Rvwbbv/g‡b bvB** Don’t know/Can’t remember | | 97 |  |
| 406 | **Avcbvi wK †Wwjfvwi e¨vM/wKU wQj?**  Did you have a delivery bag / birth kit? | **nu¨v** Yes | | 1 |  |
|  |  | **bv** No | | 2 | 🡪408 |
|  |  | **Rvwbbv/g‡b bvB** Don’t know/Can’t remember | | 97 | 🡪408 |
| 407 | **†Wwjfvwi e¨vM/wKU †Kv_vq †c‡q‡Qb?**  Where did you get birth kit? | **KwgDwbwU wK¬wbK** Community clinic | | 01 |  |
|  |  | **BDwbqb ¯^v¯’¨ I cwievi Kj¨vY †K›`ª** (H&FWC) | | 02 |  |
|  |  | **m¨v‡UjvBU wK¬wbK** Satellite clinic | | 03 |  |
|  |  | KwgDwbwU wfwËK `¶ avÎx (CSBA) | | 04 |  |
|  |  | **Dc‡Rjv ¯^v¯’¨ Kg‡cø·** Upazila Health Complex (UHC) | | 05 |  |
|  |  | **†Rjv nvmcvZvj** District hospital | | 06 |  |
|  |  | **†gwW‡Kj K‡jR nvmcvZvj** Medical College Hospita | | 07 |  |
|  |  | **gvZ…g½j †K›`ª** MCWC / Maternity centre | | 08 |  |
|  |  | **cÖvB‡fU nvmcvZvj/ wK¬wbK** Private hospital/clinic | | 09 |  |
|  |  | **Gb wR I wK¬wbK** NGO clinic | | 10 |  |
|  |  | **¯^v¯’¨ †mweKv (eªvK)** BRAC Health volunteer | | 11 |  |
|  |  | **wUweG (`vB)** Traditional Birth Attendant (TBA) | | 12 |  |
|  |  | **‡`vKvb** Shop | | 13 |  |
|  |  | **Ab¨vb¨** (wbw`©ó Kiæb) Other: | | 96 |  |
|  |  | **Rvwbbv/g‡b bvB** Don’t know/Can’t remember | | 97 |  |
| 408 | **Avcbvi †Wwjfvwii mgq †Kvb mgm¨v ev RwUjZv n‡qwQj?**  During the time of the birth of (Name) or abortion did you have any problem or complication?  **(cÖ‡Z¨KwU mgm¨v/RwUjZv m¤ú‡K© wR‡Ám Kiæb)**  (Ask all of the problems/complications) |  | **Yes** | **No** |  |
|  |  | mgm¨v/RwUjZv Problem/Complication |  |  |  |
|  |  | ev”Pv nIqvi iv¯Zv w`‡q AwZwi³ i³ wM‡qwQj Excessive Vaginal Bleeding | 1 | 2 |  |
|  |  | `yM©Ühy³ mªve wM‡qwQj Foul-Smelling Discharge | 1 | 2 |  |
|  |  | Zxeª R¡i n‡qwQj High Fever | 1 | 2 |  |
|  |  | wkïi nvZ cv Av‡M †ei n‡q G‡mwQj Baby’s Hand or Feet Coming out First | 1 | 2 |  |
|  |  | (†c‡Ui g‡a¨) wkïi A¯^vfvweK Ae¯’vb wQj Baby is in abnormal position | 1 | 2 |  |
|  |  | `xN© cÖme (12 N›Uvi †ewk) e¨_v wQj Prolong Labor (>12 hours) | 1 | 2 |  |
|  |  | cvwb †f‡½‡Q wKš‘ e¨v_v D‡Vwb Membrane ruptured but no labour pain | 1 | 2 |  |
|  |  | cøv‡m›Uv ev dzj c‡o wb Retained Placenta | 1 | 2 |  |
|  |  | ev”Pv _vKvi _wj ev Mf©`vbx ev Rš§ Øvi wQu‡o wM‡qwQj Rupture uterus/Cervical tear/Vaginal tear | 1 | 2 |  |
|  |  | (wkïi) bvox †ewi‡q G‡mwQj Cord Prolapse | 1 | 2 |  |
|  |  | (wkïi Mjvq) bvox †cuwP‡q wM‡qwQj Cord around neck | 1 | 2 |  |
|  |  | wLuPzbx n‡qwQj Convulsion | 1 | 2 |  |
|  |  | Zxeª gv_v e¨_v n‡qwQj Severe headache | 1 | 2 |  |
|  |  | ev”Pv nIqvi iv¯Zv w`‡q meyRvf wKQy †ei n‡qwQj Greenish vaginal discharge | 1 | 2 |  |
|  |  | cv/gyL dz‡j wM‡qwQj Swelling of feet or face | 1 | 2 |  |
|  |  | AviI †Kvb mgm¨v n‡qwQj Any other problem (specify)  ________________________________ | 1 | 2 |  |
| 409 | **mv¶vZKviMÖnbKvixt cÖkœ** 408 **‡`Lyb Ges mwVK †KvW e„ËvwqZ Kiæb|** Interviewer: Check Question 408 and circle appropriate code. | **GK ev GKvwaK †KvW** 1 **e„ËvwqZ** One or more codes circled | | 1 |  |
|  |  | **me¸‡jv †KvW** 2 **e„ËvwqZ** All codes circled | | 2 | 🡪500 |
| 410 | **GB mgm¨v/RwUjZvi Rb¨ Avcwb †Kvb wPwKrmv Kwi‡q‡Qb wK?**  Did you seek treatment for this complication? | **nu¨v** Yes | | 1 |  |
|  |  | **bv** No | | 2 | 🡪500 |
|  |  | **Rvwbbv/g‡b bvB** Don’t know/Can’t remember | | 97 | 🡪500 |
| 411 | **Avcwb Kvi Kvi KvQ †_‡K wPwKrmv wb‡q‡Qb?**  Whom did you see?  **DËi c‡o †kvbv‡eb bv|**  Do not read out the answers  **wR‡Ám Kiæbt AviI wKQy?**  ASK: Anything else?  **me DË‡ii †KvW e„ËvwqZ Kiæb|**  Circle code of all the answers | **¯^v¯’¨ †ckvRxwet Health personnel:** | |  |  |
|  |  | **cvk Kiv Wv³vi** MBBS doctor (Qualified) | | A |  |
|  |  | bvm©/ avÎx /c¨viv‡gwWK Nurse/Midwife/Paramedic | | B |  |
|  |  | cwievi Kj¨vY cwi`wk©Kv Family Welfare Visitor (FWV) | | C |  |
|  |  | wPwKrmv mnKvix / DcmnKvix KwgDwbwU wPwKrmv Kg©KZ©v Medical Assistant / SACMO | | D |  |
|  |  | KwgDwbwU wfwËK `¶ avÎx (CSBA) | | E |  |
|  |  | KwgDwbwU wK¬wbK wfwËK ¯^v¯’¨ †mev cÖ`vbKvix (CHCP) | | F |  |
|  |  | ¯^v¯’¨ mnKvix Health Assistant (HA) | | G |  |
|  |  | cwievi Kj¨vY mnKvix Family Welfare Assistant (FWA) | | H |  |
|  |  | Ab¨vb¨ e¨w³t Other person: | |  |  |
|  |  | **cÖwk¶YcÖvß wUweG** Trained TBA | | I |  |
|  |  | **AcÖwkw¶Z wUweG** Untrained TBA | | J |  |
|  |  | **AcÖwkw¶Z Wv³vi (MÖvg Wv³vi/ cjøx wPwKrmK/‡nvwgIc¨v_/ KweivR)** Unqualified doctor | | K |  |
|  |  | **Gb wR I ¯^v¯’¨Kg©x** NGO worker | | L |  |
|  |  | **cwiev‡ii m`m¨/AvZœxq** Family member / Relative | | M |  |
|  |  | **cÖwZ‡ekx/eÜz** Neighbour / Friend | | N |  |
|  |  | **Ab¨vb¨** (wbw`©ó Kiæb) Other: | | X |  |
|  |  | **Rvwbbv/g‡b bvB** Don’t know/Can’t remember | | Y |  |
| 412 | **GB wPwKrmv Avcwb †Kv_vq Kwi‡q‡Qb?**  Where did you seek treatment for the complication?  **me DË‡ii †KvW e„ËvwqZ Kiæb|**  Circle code of all the answers | **evwo‡Z** At home | | A |  |
|  |  | **miKvix †m±it Govt. Sector:** | |  |  |
|  |  | **nvmcvZvj/†gwW‡Kj K‡jR** Hospital/Medical college | | B |  |
|  |  | **¯^v¯’¨ I cwievi Kj¨vY †K›`ª** H&FWC | | C |  |
|  |  | **Dc‡Rjv ¯^v¯’¨ Kg‡cø·** Upazila Health Complex | | D |  |
|  |  | **m¨v‡UjvBU wK¬wbK** Satellite clinic/ EPI centre | | E |  |
|  |  | **gvZ…g½j †K›`ª** MCWC / Maternity centre | | F |  |
|  |  | **cwievi Kj¨vY mnKvix** FWA | | G |  |
|  |  | **KwgDwbvU `¶ avÎx** CSBA | | H |  |
|  |  | **Kgy¨DwbwU wK¬wbK** Community clinic | | I |  |
|  |  | **Ab¨vb¨** (wbw`©ó Kiæb) Other:_________________ | | J |  |
|  |  | Gb wR I ‡m±it NGO Sector: | |  |  |
|  |  | **Gb wR I ¯’vqx †K›`**ª NGO Static Clinic | | K |  |
|  |  | **Gb wR I A¯’vqx †K›`**ª NGO Satellite Clinic | | L |  |
|  |  | **Ab¨vb¨** (wbw`©ó Kiæb) Other: | | M |  |
|  |  | **cÖvB‡fU †gwW‡Kj †m±it Private Medical Center:** | |  |  |
|  |  | **cÖvB‡fU wK¬wbK/nvmcvZvj** Private clinic/Hospital | | N |  |
|  |  | **cvkKiv Wv³vi** Qualified doctor (MBBS) | | O |  |
|  |  | **AcÖwkw¶Z Wv³vi (MÖvg Wv³vi/KweivR)** Unqualified doctor | | P |  |
|  |  | **dv‡g©mx** Pharmacy | | Q |  |
|  |  | **Ab¨vb¨** (wbw`©ó Kiæb) Other: | | R |  |
|  |  | Ab¨vb¨ Other sources: | |  |  |
|  |  | **‡`vKvb** Shop | | S |  |
|  |  | **eÜz/AvZ¥xq** Friends/Relatives | | T |  |
|  |  | **wUweG / `vB** TBA | | U |  |
|  |  | **Ab¨vb¨** (wbw`©ó Kiæb) Other | | X |  |

**ধন্যবাদ।**

**Thank you for participation.**
